# Unique Features of Network Bursts Emerge From the Complex Interplay of Excitatory and Inhibitory Receptors in Rat Neocortical Networks

**DOI:** 10.3389/fncel.2019.00377

**Published:** 2019-09-06

**Authors:** Heidi Teppola, Jugoslava Aćimović, Marja-Leena Linne

**Affiliations:** Computational Neuroscience Group, Faculty of Medicine and Health Technology, Tampere University, Tampere, Finland

**Keywords:** AMPA receptor, cell culture, GABA_A_ receptor, neocortical cells, neuronal network, NMDA receptor, microelectrode array, spontaneous network burst activity

## Abstract

Spontaneous network activity plays a fundamental role in the formation of functional networks during early development. The landmark of this activity is the recurrent emergence of intensive time-limited network bursts (NBs) rapidly spreading across the entire dissociated culture *in vitro*. The main excitatory mediators of NBs are glutamatergic alpha-amino-3-hydroxy-5-methyl-4-isoxazolepropionic acid receptors (AMPARs) and *N*-Methyl-D-aspartic-acid receptors (NMDARs) that express fast and slow ion channel kinetics, respectively. The fast inhibition of the activity is mediated through gamma-aminobutyric acid type A receptors (GABA_A_Rs). Although the AMPAR, NMDAR and GABA_A_R kinetics have been biophysically characterized in detail at the monosynaptic level in a variety of brain areas, the unique features of NBs emerging from the kinetics and the complex interplay of these receptors are not well understood. The goal of this study is to analyze the contribution of fast GABA_A_Rs on AMPAR- and NMDAR- mediated spontaneous NB activity in dissociated neonatal rat cortical cultures at 3 weeks *in vitro.* The networks were probed by both acute and gradual application of each excitatory receptor antagonist and combinations of acute excitatory and inhibitory receptor antagonists. At the same time, the extracellular network-wide activity was recorded with microelectrode arrays (MEAs). We analyzed the characteristic NB measures extracted from NB rate profiles and the distributions of interspike intervals, interburst intervals, and electrode recruitment time as well as the similarity of spatio-temporal patterns of network activity under different receptor antagonists. We show that NBs were rapidly initiated and recruited as well as diversely propagated by AMPARs and temporally and spatially maintained by NMDARs. GABA_A_Rs reduced the spiking frequency in AMPAR-mediated networks and dampened the termination of NBs in NMDAR-mediated networks as well as slowed down the recruitment of activity in all networks. Finally, we show characteristic super bursts composed of slow NBs with highly repetitive spatio-temporal patterns in gradually AMPAR blocked networks. To the best of our knowledge, this study is the first to unravel in detail how the three main mediators of synaptic transmission uniquely shape the NB characteristics, such as the initiation, maintenance, recruitment and termination of NBs in cortical cell cultures *in vitro*.

## Introduction

Spontaneous network activity plays a fundamental role in the formation of functional networks during early development of the central nervous system (Feller, 1999; O’Donovan, 1999; Ben-Ari, 2001; Blankenship and Feller, 2010; Egorov and Draguhn, 2013; Luhmann et al., 2016). Recurrent network bursts (NBs) are observed in cerebral cortex *in vivo* (Chiu and Weliky, 2001; Crochet et al., 2005; Minlebaev et al., 2007; Yang et al., 2009; Wang and Arnsten, 2015), in cortical brain slice preparations *in vitro* (Yuste and Katz, 1991; Garaschuk et al., 2000; Harsch and Robinson, 2000; Sanchez-Vives and McCormick, 2000; Corner et al., 2002; Corlew et al., 2004; Sun and Luhmann, 2007; Allene et al., 2008), as well as in dissociated *in vitro* cortical cell cultures (Dichter, 1978; Murphy et al., 1992; Muramoto et al., 1993; Maeda et al., 1995; Marom and Shahaf, 2002; Opitz et al., 2002; van Pelt et al., 2004; Chiappalone et al., 2006; Eytan and Marom, 2006; Wagenaar et al., 2006; Tetzlaff et al., 2010; Teppola et al., 2011; Weihberger et al., 2013; Okujeni et al., 2017). Considering that the bursting dynamics are an essential feature of the activity both *in vivo* and *in vitro* and that the complex underlying mechanisms that shape this activity are not well understood, their better characterization is highly important. Since *in vitro* networks are easy to control under several pharmacological conditions with simultaneous long-term recordings of the evolution of the extracellular network-wide activity with microelectrode arrays (MEAs), they provide a model system that ultimately helps to unravel and compare the receptor mechanisms that modulate the dynamics of cortical circuits *in vivo*. In this work we study how the spontaneous NBs are shaped by the underlying interplay of the excitatory and inhibitory receptors of synaptic transmission to modify excitation-inhibition balance in dissociated *in vitro* cultures extracted from both hemispheres of postnatal (P0) rats. The goal of the study is to analyze the contribution of fast gamma-aminobutyric acid type A receptors (GABA_A_Rs) on alpha-amino-3-hydroxy-5-methyl-4-isoxazolepropionic acid receptor- (AMPAR-) and N-methyl-D-aspartate receptor- (NMDAR-) mediated spontaneous NB activity at 3 weeks *in vitro* cultures. The importance of the systematic analysis of data is emphasized throughout the study to enable efficient comparison of data obtained from different *in vitro* networks and the validation of future computational models.

In mature cortical cell cultures, spontaneous NBs consist of ∼0.5 s long period of extensive spiking across the culture separated by ∼7 s long silent steady interburst intervals (IBIs) (Kuroda et al., 1992; Muramoto et al., 1993; Robinson et al., 1993; Teppola et al., 2011). These NBs are shown to be driven by the excitatory synaptic transmission (Robinson et al., 1993; Jimbo et al., 2000) which is primarily mediated by the action of glutamate on two types of glutamatergic ionotropic receptors, AMPARs and NMDARs (Hollmann and Heinemann, 1994; Dingledine et al., 1999). It has been shown with patch-clamp technique that zero magnesium (Mg^2+^) in the medium results in large slow excitatory postsynaptic currents (EPSCs) that occur simultaneously with the NB depolarization (Robinson et al., 1993). The NB depolarization is reversibly inhibited either with 100 μM NMDAR antagonist 2-amino-5-phosphovaleric acid (APV) or with 2 mM Mg^2+^, producing large hyperpolarization in cortical cultures (Robinson et al., 1993). This inhibition indicates that the NMDAR-mediated component of EPSC is essential for the NB activity and that the NMDARs are tonically active at rest before inhibition. In addition, periodic activity can be blocked with 30 μM AMPAR antagonist 6-cyano-7-nitroquinoxaline-2,3-dione (CNQX), implying that there is also a significant AMPA component in the synaptic currents (Robinson et al., 1993). When the cultures are electrically stimulated, the evoked responses of the cortical cells are shown to be composed of the early and the late phases, which are produced by the activation of distinct synaptic pathways (Jimbo et al., 2000; Marom and Shahaf, 2002; Wagenaar et al., 2004). The early phase component includes early post-synaptic spikes that occur between 5 and 50 ms after the stimuli, and their temporal precision varies around 2 ms with low reliability (Wagenaar et al., 2004). The NMDARs are involved in the generation of the late phase since the time constant of late-phase (160 ms) is in the same order of magnitude as the decay time of NMDAR-mediated EPSC (Jimbo et al., 2000). Here, we start by showing that AMPARs play a greater role in the initiation of spontaneous NBs, while NMDARs are maintaining the already initiated activity in post-natal rat cortical dissociated cultures *in vitro*.

Inhibitory synaptic transmission is known to be mediated by the action of gamma-aminobutyric on gabaergic receptors (GABARs) in these networks (Ramakers et al., 1990; Baltz et al., 2010). GABAergic signaling and interneurons are crucial in the spontaneous network activity of a developing neocortex (Moody and Bosma, 2005; Ben-Ari et al., 2007). Depolarizing GABAergic signaling has been implicated in the early morphological and functional maturation of neuronal networks (Owens and Kriegstein, 2002; Cancedda et al., 2007; Pfeffer et al., 2009; Wang and Kriegstein, 2011). The developmental GABA shift from depolarizing to hyperpolarizing signaling represents a hallmark in cortical network maturation. This shift closes the period of GABAR-driven network oscillations and sets the onset of mature GABAergic function (Rivera et al., 2005; Ben-Ari et al., 2007). The developmental alterations of the network activity and their correlates with the GABA shift are also shown in dissociated cortical cultures (Soriano et al., 2008; Baltz et al., 2010). Previous research has shown that less synchronized burst activity correlates with the gradual maturation of GABA_A_ receptor signaling, which depends on the presence of large GABAergic neurons with widespread connections in cultured cortical networks (Baltz et al., 2010). Additionally, it has been demonstrated that the late phase substantially increases after the blockade of GABA_A_Rs with their antagonists (10μM bicuculline (BIC), 5μM picrotoxin (PTX) or 20μM gabazine) which indicates that the intensity and duration of the late phase are controlled by inhibitory synapses among cortical neurons *in vitro* (Jimbo et al., 2000; Weihberger et al., 2013; Baltz and Voigt, 2015). Furthermore, GABAergic interneurons are shown to control the dynamic spatio-temporal pattern formation in neuronal networks by organizing spatially and temporally the network activity rather than only reducing firing probability (Whittington and Traub, 2003; Mann and Paulsen, 2007; Klausberger and Somogyi, 2008).

Although the excitatory and inhibitory synaptic transmission including the kinetics of AMPAR, NMDAR, and GABA_A_R are biophysically well characterized at the monosynaptic level, their complex interplay and contribution to network activity dynamics are not well understood. Specifically, it is not known how GABA_A_Rs separately shape AMPAR- and NMDAR-mediated NB dynamics. In this work, we study the contribution of fast AMPAR-, and slow NMDAR-mediated recurrent excitatory signaling and the contribution of fast GABA_A_R-mediated inhibitory signaling to initiate, maintain, propagate and terminate NBs. Our focus is on how inhibitory GABA_A_Rs individually shape the AMPAR- or NMDAR-mediated spontaneous network activity dynamics. To identify these receptor mechanisms and to determine how GABA_A_Rs modify AMPAR- and NMDAR-mediated spontaneous NB dynamics, the extracellular activity was systematically recorded using the MEA technique under several combinations of receptor antagonists (see I–V) in P0 neocortical cultures at the end of the 3rd week *in vitro*. In addition, the recorded multiunit-spike data was quantitatively analyzed (see 1–11). In brief, the following five conditions were studied: I mature control (CTRL) cultures without pharmacology, II cultures with AMPAR blockade (NBQX, 1,2,3,4-Tetrahydro-6-nitro-2,3-dioxo-benzo[f]quinoxaline-7-sulfonamide disodium salt hydrate), i.e., NMDAR-mediated synaptic transmission, III cultures with NMDAR blockade (D-AP5, D-2-Amino-5-phosphonovaleric acid), i.e., AMPAR-mediated synaptic transmission, IV disinhibited cultures from II with GABA_A_R blockade (PTX), and V disinhibited cultures from III with the GABA_A_R blockade (PTX). These five conditions were used in two different types of experimental protocols. In the first, neurons were initially probed by acute application of a high concentration of either the AMPAR or NMDAR antagonist and then by acute application of a high concentration of the GABA_A_R antagonist. In the second, an increasing concentration of either AMPAR or NMDAR antagonist was gradually applied, thus allowing neurons to adapt to the reduced synaptic transmission of each specific receptor type. We analyzed the multi-unit spike data with our focus being on the (1) overall firing rate (OFR [Hz]), (2) burst frequency (BF [NB/min]), (3) burst length (BL [s]), (4) falling phase (FP [s]), (5) rising phase (RP [s]), (6) maximum firing rate within the NB (MFR [Hz]), (7) burst size (BS [spikes]), and (8) electrode recruitment count (RC). We also analyzed (9) interspike intervals (ISI [s]) and (10) interburst intervals (IBI [s]) as well as (11) the network recruitment time of active electrodes at the beginning of the NBs and (12) the similarity of spatio-temporal patterns of NBs. The systematic analysis enables the efficient comparison of data and the validation of future computational models.

In this study, we show that the networks express NBs when either AMPARs or NMDARs are acutely blocked with as high a concentration as 10 μM of NBQX or D-AP5. We present that AMPARs facilitate the fast initiation and fast recruitment of neurons into the network activity and further increase the diversity of spatio-temporal patterns. Our results also confirm that NMDARs contribute temporally and spatially to maintain the highly repetitive spatio-temporal patterns. Furthermore, the results show that GABA_A_Rs reduce the spiking frequency and coverage as well as slow down the recruitment of neurons into the network activity. In particular, GABA_A_Rs prevented the spiking frequency in AMPAR-mediated networks and dampened the termination of NBs in NMDAR-mediated networks. In addition, GABA_A_Rs further increased the diversity of spatio-temporal patterns in NMDAR-mediated networks. To the best of our knowledge, this study is the first to unravel in detail how the three main mediators of synaptic transmission uniquely shape the NB characteristics, such as the initiation, maintenance, propagation and termination of NBs in newborn rat dissociated neocortical cell cultures *in vitro*. Additionally, the study supports developing new computational models of cortical *in vitro* networks to understand their dynamics both in healthy and pathological conditions.

## Materials and Methods

We conducted a re-analysis of previously published results (Teppola et al., 2011), significantly expanded the set of employed data analysis methods and obtained entirely new findings using these methods. We particularly focused on the systematic quantitative characterization of various aspects of spontaneous network activity to support the future development of computational models.

### Preparation and Maintenance of Cell Cultures

**FIGURE 1:**
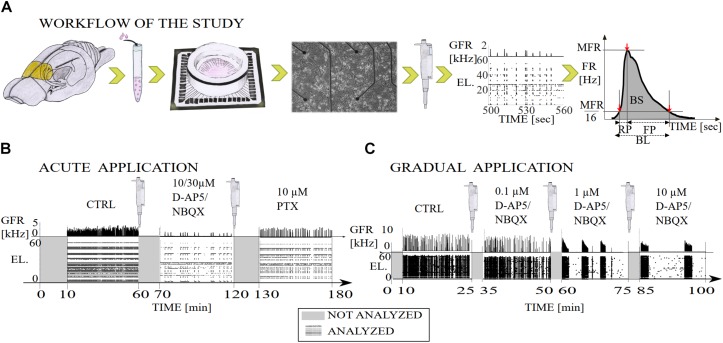
Illustration of the workflow of the study and diagrams of the experimental recording protocols with pharmacological procedures. **(A)** The primary cell cultures were extracted from the prefrontal cortex of the postnatal brains of Wistar rats (yellow). The cortical cells were derived from the tissue by mechanical chopping, trypsinization and dissociation, the cells were seeded on polyethyleneimine coated MEA dishes and cultured until 3 weeks *in vitro*. Network-wide activity was recorded with MEA before and after either the gradual or acute application of excitatory or inhibitory synaptic receptor antagonists. Data analysis was performed on the multi-unit spike times, including NB detection, computation of the burst measures, such as BL, RP, FP, MFR, BS, and RC. Additionally, we computed interspike interval distributions (ISI), interburst-interval distributions (IBI) and cumulative time curves of electrode recruitment (see section “Materials and Methods”). **(B)** Timeline of a total of 180 min of recording in which excitatory synaptic transmission was probed by acute application of either AMPAR or NMDAR antagonist (NBQX and D-AP5, respectively both 10 μM), and inhibitory synaptic transmission was probed by acute application of GABA_A_R antagonist (PTX, 10 μM). After application of an antagonist the first ten minutes of the recording were not analyzed and the remaining 50 min of the recording were analyzed. **(C)** Timeline of a total of 100 min of recording in which excitatory synaptic transmission was probed by gradual application of AMPAR (NBQX, 0.1, 1, and 10 μM) or NMDAR (D-AP5 0.1, 1, and 10 μM) antagonist. The first ten minutes of the recording were not analyzed due to the transition phase (gray) and the remaining 15 min were analyzed (raster plot with global firing rate [kHz]). GFR denotes the global firing rate and EL the electrode numbers.

In Teppola et al. (2011), the primary cortical cell cultures were prepared as described previously (Shahaf and Marom, 2001), and animal handling and tissue preparation were performed in accordance with guidelines for animal research at the University of Freiburg. No further preparation of cell cultures were required for the present study. The cells were derived from the prefrontal cortex of both hemispheres (Figure 1A) of postnatal (P0) Wistar rat pups of either sex. Cortical tissue was mechanically chopped with a scalpel in cold phosphate-buffered saline solution (PBS, all reagents from GIBCO, Invitrogen, Karlsruhe, Germany, unless otherwise stated) and enzymatically treated with 0.05% trypsin solution in a shaker for 15 min at 37°C. Trypsinization was stopped with 20% horse serum. DNase (Sigma-Aldrich, Steinheim, Germany) was added (type IV 50 μg/ml) to degrade deoxyribonuclease around cells. Cells were dissociated by gentle trituration with a serological pipette (10 ml; Becton Dickinson, Franklin Lakes, NJ, United States) in PBS, centrifuged (5 min 2000rcf, Rotofix 32A, Hettich, Tuttlingen, Germany) and re-suspended into minimum essential medium (MEM, supplemented with 5% heat-inactivated horse serum, 20 mM glucose, 0.5 mM L-glutamine, and 1% gentamicine, 1 ml MEM/rat). Cells were counted with an automated cell counter (CASY TT, Schärfe Systems GmbH, Reutlingen, Germany) and seeded at densities of 2,000 cells/mm^2^ onto polyethyleneimine (PEI, 200 μl, 0,2%, Sigma-Aldrich, Steinheim, Germany) coated MEAs (MultiChannel Systems Ltd., Reutlingen, Germany). Cells were cultured in 2 ml MEM in 5% CO_2_ humidified incubator at 37°C. One third of the medium was changed twice a week and recordings were done between 20–25 days *in vitro* (DIV). All the cultures were carefully prepared and validated in order to provide as stable and comparable recording conditions as possible for each protocol and culture. In particular, the cell density (2000 cells/mm^2^) and the maturation point (20–25 DIV) were selected according to the literature (Wagenaar et al., 2006) to guarantee appropriate experimental model system to be combined with the standard MEA.

### Pharmacology

In Teppola et al. (2011), the AMPAR-mediated ionotropic glutamatergic transmission was probed by competitive antagonist of the AMPARs 1,2,3,4-Tetrahydro-6-nitro-2,3-dioxo-benzo[f]quinoxaline-7-sulfonamide disodium salt hydrate (NBQX). The NMDAR-mediated ionotropic glutamatergic transmission was probed by competitive antagonist of the glutamate site of the NMDARs D-(-)-2-Amino-5-phosphonopentanoic acid (D-AP5). The ionotropic GABAergic transmission was probed by non-competitive GABA_A_R antagonist picrotoxin (PTX). These reagents were used for suppressing or blocking the function of each receptor type, allowing to study the contribution of the receptors for network activity dynamics during electrophysiological recordings. All drugs were directly applied with a pipette into the culture medium inside the dry incubator. All pharmacological chemicals were purchased from Sigma-Aldrich, Steinheim, Germany.

Synaptic transmission was probed by both acute and gradual applications of AMPAR and NMDAR antagonists and by acute application of GABA_A_R antagonist. In the case of acute antagonist applications, higher concentrations of the drug were applied to simultaneously block all specific types of receptors in the whole cell culture. Acute application enabled the study of the acute influence of an antagonist, i.e., to induce the complete blockade of each receptor type on network activity dynamics. In the case of gradual antagonist applications, increasing concentrations of the drug were applied to the culture, thereby allowing neurons to briefly adapt to the reduced synaptic transmission of each specific receptor type. Gradual application enabled the study of the concentration-dependent effects of each excitatory antagonist on network dynamics.

**FIGURE 2:**
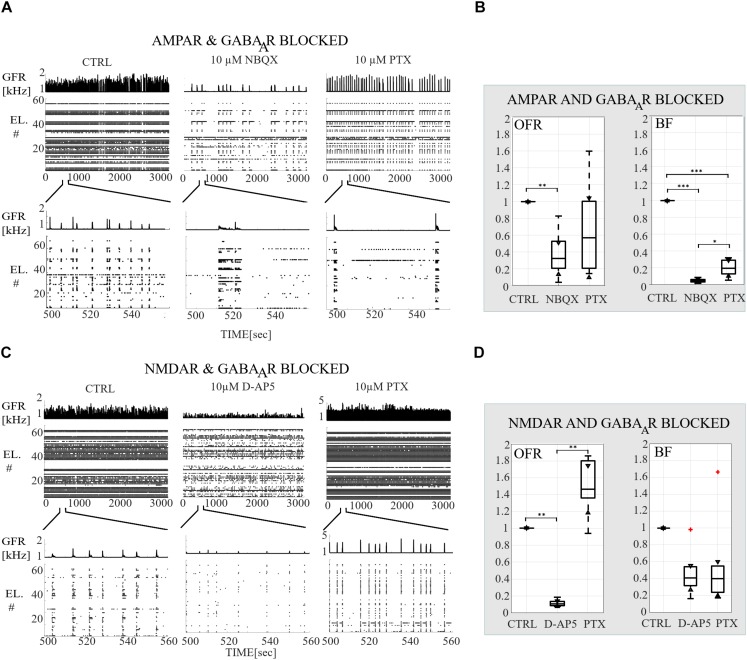
Receptor-dependent network-wide activity probed by acute application of AMPA, NMDA and GABA_A_ receptor antagonists, relative changes in OFR and in BF. **(A)** Raster plots of network-wide activity. In each subpanel, the global firing rates (GFR [kHz]) are displayed on top of raster plots of spike times (TIME [s]) from each electrode (EL #) in each condition. The first subpanel shows the CTRL recording, the second subpanel the activity after acute application of AMPAR antagonist (10 μM NBQX), and the third subpanel the activity after acute applications of both antagonists of AMPAR and GABA_A_R (10 μM PTX). The top row shows the complete analyzed recording of 3,000 s, and the bottom row enlargements of 60 s to display fine details of the activity. **(B)** Relative change in OFR and in BF are computed for each recording and then displayed as box plot representation of pooled rates of the CTRL recordings (*n* = 7), of the recordings when AMPARs are antagonized (NBQX, *n* = 7) and of the recordings when both AMPARs and GABA_A_Rs (PTX, *n* = 7) are antagonized. **(C)** Same as **(A)** except that the CTRL recording is compared to the recording when NMDARs (10 μM D-AP5) and both NMDARs with GABA_A_Rs (10 μM PTX) are acutely antagonized. **(D)** Same as **(B)** except that the CTRL recordings (*n* = 6) are compared to conditions when NMDARs are antagonized (D-AP5, *n* = 6) and to the conditions when both NMDARs and GABA_A_Rs (PTX, *n* = 6) are antagonized. Both AMPAR and NMDAR antagonists significantly decreased the OFR (*p*_ranksum_ < 0.01) **(B,D)**. Disinhibition significantly increased the OFR in NMDAR blocked cultures (*p*_ranksum_ < 0.01), but not in AMPAR blocked cultures compared to the condition before disinhibition **(B,D)**. AMPAR blockade significantly decreased the network BF (*p*_ranksum_ < 0.001) and followed disinhibition slightly increased BF (*p*_ranksum_ < 0.05) compared to the condition before disinhibition. The box plots show the median, 25^th^ and 75^th^ percentiles and whiskers extending to the minimal and maximal values and the plus signs represent outliers. ^∗∗∗^*p*_ranksum_ < 0.001, ^∗∗^*p*_ranksum_ < 0.01, ^∗^*p*_ranksum_ < 0.05.

The AMPARs were acutely antagonized with NBQX that was applied at concentrations completely blocking the AMPAR-mediated ionotropic glutamatergic transmission (for 10 μM, see Figure 2A and Table 1, Protocol id 1, and for 30 μM, see Supplementary Figure S1C and Supplementary Table S1). The concentration of 10 μM of NBQX has been shown to be sufficient to block the AMPA receptor mediated current (Parsons et al., 1994). The NMDARs were acutely antagonized with D-AP5 that was applied at concentrations of 10 or 30 μM (for 10 μM, see Figure 2C and Table 1, Protocol id 2, and for 30 μM, see Supplementary Figure S1C and Supplementary Table S1). Previous literature has shown that 10 μM of D-AP5 is sufficient to block 95% of the NMDA receptor mediated currents (Benveniste and Mayer, 1991). Additionally, we performed experiments where 30 μM of D-AP5 or NBQX was used to be confident that AMPA or NMDA receptor mediated currents were completely antagonized (see Supplementary Material section). The results were consistent with both concentrations (10 and 30 μM D-AP5 or NBQX). Therefore, we show in the manuscript only the results with 10 μM NBQX and in the Supplementary Material section the results with 30 μM. Cultures were disinhibited and GABA_A_Rs acutely antagonized with PTX applied at a concentration of 10 μM which is shown to completely block ionotropic GABAergic transmission (Krishek et al., 1996) (Figures 1B, 2A,C and Table 1, Protocol id 1, 2).

**TABLE 1:** List of experimental protocols including recording conditions, blocked and functional receptors and the number of used cultures.

**Protocol id**	**Recording condition/Drug**	**Concentration of an antagonist**	**Blocked receptors**	**Functional receptors among the considered ones**	**Number of cultures**
**Acute application**					
1	CTRL	–	None	All	7
	NBQX	10 μM	AMPA	NMDA, GABA_A_	
	PTX	10 μM	GABA_A_	NMDA	
2	CTRL	–	None	All	6
	D-AP5	10 μM	NMDA	AMPA, GABA_A_	
	PTX	10 μM	GABA_A_	AMPA	
**Gradual application**					
3	CTRL	–	None	All	2
	NBQX	0.1, 1, 10 μM (in total 11.1 μM)	AMPA	NMDA, GABA_A_	
4	CTRL	–	None	All	2
	D-AP5	0.1, 1, 10 μM (in total 11.1 μM)	NMDA	AMPA, GABA_A_	

In the case of gradual application of the AMPAR antagonist, an increasing amount of NBQX was applied to first partially (0.1 and 1 μM) and then completely block the AMPAR-mediated transmission (10 μM, Parsons et al., 1994) (Figures 1C, 6A and Table 1, Protocol id 3). In the gradual application of the NMDAR antagonist, the selected increasing amount of D-AP5 (0.1, 1, and 10 μM) was applied to gradually reduce the functioning of NMDAR-mediated ionotropic glutamatergic transmission (Benveniste and Mayer, 1991) (Figures 1C, 6C and Table 1, Protocol id 4).

### Experimental Protocols

A total of 20 cell cultures from seven different preparations were studied by recording the network activity of the cultures with MEAs. In the case of acute application of the excitatory receptor antagonists (AMPAR or NMDAR blockade) followed by the acute application of the inhibitory GABA_A_R antagonist, a total of 13 cultures from three different preparations were studied. Seven cultures were used for experiments of acute AMPAR blockade (10 μM NBQX) followed by acute GABA_A_R blockade (10 μM PTX). Six cultures were used for experiments of acute NMDAR blockade (10 μM D-AP5) followed by acute GABA_A_R blockade (10 μM PTX). Each experiment consisted of the following steps: (1) one hour long CTRL recording, (2) application of 10 μM antagonist (NBQX in experiments with AMPAR blockade, D-AP5 in experiments with NMDAR blockade), (3) recording for one hour, (4) application of 10 μM PTX, and (5) recording for 1 h (Figures 1B, 2A,C and Table 1, Protocol id 1, 2). After the application of the antagonist, the first ten minutes of the recording were not analyzed to avoid transition phases. The remaining 50 min of the recording were analyzed.

In the case of gradual application of the excitatory receptor antagonists (AMPAR or NMDAR blockade), a total of four cell cultures from two different preparations were studied. Two cultures were used for experiments with gradual AMPAR blockade (0.1, 1, and 10 μM NBQX) and two cultures for experiments with gradual NMDAR blockade (0.1, 1, and 10 μM D-AP5). Each experiment consisted of the following steps: (1) 25 min-long CTRL recording, (2) application of 0.1 μM antagonist (NBQX in experiments with AMPAR blockade, D-AP5 in experiments with NMDAR blockade), (3) recording for 25 min, (4) application of 1 μM antagonist, (5) recording for 25 min, (6) application of 10 μM antagonist, and (7) recording for 25 min (Figures 1C, 6A,C and Table 1, Protocol id 3, 4). For each 25 min-long recording, the first 10 min were not analyzed to avoid transition phases. The remaining 15 min were analyzed as described below. A total of three cultures were used to study gradual disinhibition, see Supplementary Material section (Supplementary Table S1 and Supplementary Figures S1–S4).

### Extracellular Microelectrode Array Recording

In Teppola et al. (2011), the spontaneous extracellular bioelectrical activity was recorded from the cell cultures inside a dry incubator (37°C, 5% CO_2_). The activity was collected using MEAs (MultiChannel Systems Ltd., Reutlingen, Germany). The 59 TiN electrodes, Ø 30 μM, were placed in rectangular pattern (6 × 10 grid with 500μm pitch distance). The recorded signals were amplified using MEA-1060-BC built-in system amplifier with 25 kHz sampling frequency. The recorded data was acquired using MC-Rack software (Multi Channel Systems Ltd., versions 3.3–4.5). Raw signals from each electrode were digitally high-pass filtered at 200 Hz cutoff and with detection dead time of 2 ms (Butterworth, 2nd order high-pass filter). Finally, the signals were thresholded at −5 times of standard deviation (SD) from the mean noise baseline for each electrode. The times when the signals crossed this voltage threshold were detected as spike times for further analysis. The collection of spike times obtained from each electrode provides an estimation of NB activity, we will refer to it as NB spike times in what follows. In the case of gradual AMPAR blockade, we observed strong occurrence of superbursts (SBs), which lasted ∼100 s with 3–8 min silent steady period. Similar SBs occurring in short, sharply defined trains with several minutes of silent periods have been described previously in young cultures at 7–11 DIV (Wagenaar et al., 2006). The raw data from MEA recordings, i.e., spike times and electrode indices, were imported into MATLAB (version 2013b, The Mathworks Inc., MA, United States) using MEA-Tools (Egert et al., 2002) and the FIND-Toolbox (Meier et al., 2008). The NB detection and further advanced data analysis, statistics and image preparation were completed using our own Matlab (version 2013b, The Mathworks Inc., MA, United States) code.

### Preprocessing of Data and Burst Detection

The initial 10 min of the recording after the application of an antagonist were not analyzed to avoid transient phases. The following 15 or 50 min, depending of the recording protocol, were then analyzed (Figures 1B,C). Electrodes with firing rate (FR) lower than 9% of the average FR on electrodes with spike activity were excluded. Spontaneous NBs were detected according to the following criteria: the first interspike interval <100 ms defined the onset time of the NB and the first interspike interval >100 ms defined the offset of the NB. In addition, the following criteria were included in the NB detection algorithm: a minimum of five electrodes must be active during the NB and a minimum of five spikes must be recorded per NB. Similar burst detection criteria has been previously presented in the literature, see for example (Chiappalone et al., 2006). All data was manually inspected to verify that the bursts were correctly and as accurately as possible identified when recorded with the standard 59 TiN electrode MEA technique. Standard MEA technique is a reliable and accepted recording technique for high-throughput screening of network dynamics in multiple cultures and protocols (Wagenaar et al., 2006). In addition to the standard MEA, the high-density MEAs have been used in the literature to obtain extra high accuracy and precision when detecting random spiking, burst duration, activity propagation and NB rates (Gandolfo et al., 2010; Maccione et al., 2010; Lonardoni et al., 2015).

### Relative Overall Firing Rate and Burst Frequency Computations

The relative changes in OFR [Hz] and network BF [NB/min] were computed for each recording and then displayed as a box plot representation of pooled rates in the acute application of the excitatory/inhibitory receptor blockade (see Table 1, Protocol id 1, 2). The box plots show the median, 25^th^ and 75^th^ percentiles with whiskers extending to the minimal and maximal values. The plus signs represent outliers in Figures 2B,D. Statistical analysis was performed for pooled rates using Wilcoxon rank sum test. Differences were considered to be significant when *p*_ranksum_ < 0.05, *p*_ranksum_ < 0.01, or *p*_ranksum_ < 0.001, different significances are indicated with ^∗^, ^∗∗^, ^∗∗∗^, respectively, in Figures 2B,D.

Relative changes in OFR and BF were computed for each recording and then displayed as separate graph representations of the rates in the cases of gradual application of the excitatory receptor blockade (see Table 1, Protocol id 3, 4, Figures 6B,D, and Supplementary Figures S1B,D). To compute the relative OFR, the number of all spikes was divided by the duration of the recording period in seconds. To compute the BF, the number of NBs was divided by the duration of the recording period in minutes. The values were normalized to CTRL condition by dividing the value with the CTRL value.

### Characterization of Burst Measures

To analyze the characteristic burst measures, NB firing rate (FR [Hz]) profiles were calculated for each NB. NB rate profiles were calculated by smoothing the spike counts in the bins via convolution with a Gaussian kernel (SD of 15). NB measures were calculated from NB rate profiles as follows; (1) MFR [Hz] was defined as the peak of the profile, (2) the RP [s] starts at the time point when the FR reaches 1/16^th^ of the MFR and ends when the FR reaches the time point of the MFR, (3) the FP [s] starts at the time point of MFR and ends when FR decreases back to 1/16^th^ of the MFR, and (4) BL [s] is the sum of RP and FP (Figure 1A). In addition, we calculated (5) the BS [spikes], i.e., number of spikes per each NB, and (6) RC, i.e., the number of active electrodes during the NB. BS and RC were calculated from the start time point until the end time point of the detected NBs. We calculated the NB measures i.e., the peak of NB and the intervals between the time of the peak of NB and the time at 1/16^th^ of the height of NB similar to Gritsun et al. (2010). The method reduces the variability and possible oscillating tails of NBs and also provides a systematic measure for comparing NB rate profiles under different pharmacology. Similar burst measures have been previously presented in the literature, see for example (Vajda et al., 2008; Bologna et al., 2010). However, the set of experimental protocols, burst measures and analysis tools in the presented work is extensive.

**FIGURE 3:**
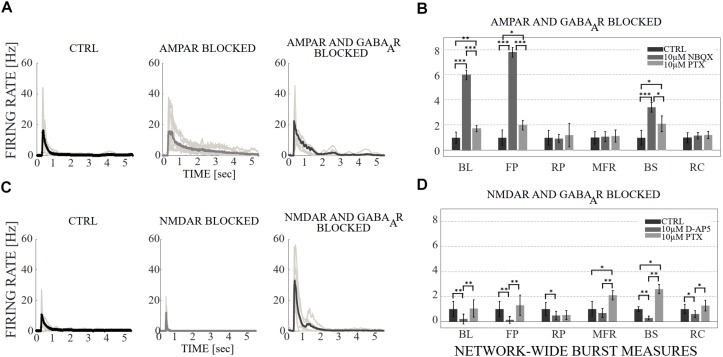
The influence of acute application of the excitatory and inhibitory receptor antagonists on NB profiles and measures. **(A)** The top row shows the NB profiles [Hz] from six congruent experiments where the data of the CTRL recording (1^st^ subpanel) is compared to the data of the recording conditions when AMPARs (2^nd^ subpanel), and AMPARs with GABA_A_Rs (3^rd^ subpanel) are antagonized. The thin gray lines represent the average NB profiles of each culture, whereas the thick line represents the average over all cultures. The profiles are aligned with each other at the time point when the rate is reached. The presented profiles are computed using the spike-data from Figure 2, processed as described in section “Materials and Methods”. **(B)** The relative change of characteristic burst measures including, BL, FP, RP, MFR, BS and RC are extracted from NB profiles (see section “Materials and Methods”). Each bar represents the mean ± SD of the medians from all cultures. The values were normalized to the CTRL condition. The panel shows the relative change in AMPAR (10 μM NBQX) blocked networks, followed by GABA_A_R (10 μM PTX) blockade (*n* = 7). **(C)** Shows the same as **(A)**, except the data of the CTRL recording (1^st^ subpanel) is compared to the data of the recording conditions when NMDARs (2^nd^ subpanel) and NMDARs with GABA_A_Rs (3^rd^ subpanel) are antagonized. **(D)** Shows the same as **(B)**, except that the relative change is computed in NMDAR blocked networks (10 μM D-AP5), followed by GABA_A_R (10 μM PTX) blockade (*n* = 6). BL, FP and BS increased significantly (*p*_ranksum_ < 0.001) when applying acute AMPAR blockade compared to CTRL **(B)**. BL (*p*_ranksum_ < 0.001), FP (*p*_ranksum_ < 0.001) and BS (*p*_ranksum_ < 0.05) decreased significantly when acutely disinhibited, and compared to previous AMPAR blocked condition **(B)**. In contrast, BL (*p*_ranksum_ < 0.01), FP (*p*_ranksum_ < 0.01), RP (*p*_ranksum_ < 0.05), BS (*p*_ranksum_ < 0.01) and RC (*p*_ranksum_ < 0.05) significantly decreased when NMDARs were blocked **(D)**. Furthermore, BL (*p*_ranksum_ < 0.01), FP (*p*_ranksum_ < 0.01), MFR (*p*_ranksum_ < 0.01), BS (*p*_ranksum_ < 0.01) and RC (*p*_ranksum_ < 0.05) significantly increased when disinhibited and compared to the previous NMDAR blocked condition **(D)**. ^∗∗∗^*p*_ranksum_ < 0.001, ^∗∗^*p*_ranksum_ < 0.01, ^∗^*p*_ranksum_ < 0.05.

The MFR and BS were additionally divided by the recruitment count, i.e., the number of active electrodes, to have MFR per electrode and BS per electrode. Firstly, for each NB measure (except the maximum of RC) and each culture, we computed the median across all NBs recorded in that culture. Then we computed the mean and the SD across all medians obtained from all cultures. Secondly, we divided SD by the mean to calculate the coefficient of variation, i.e., relative SD. Thirdly, we normalized the mean of medians to CTRL. Fourthly, the relative values (mean and SD) of all NB measures were displayed as bars with error bars (Figures 3B,D, 7B,D). Wilcoxon rank sum test and *p*-values were computed for all burst measures in each condition and in each culture. If the tests showed a similar result for each culture, the significance was displayed as ^∗∗∗^*p*_ranksum_ < 0.001, ^∗∗^*p*_ranksum_ < 0.01, ^∗^*p*_ranksum_ < 0.05 in Figures 3B,D, 7B,D.

### Interspike Interval and Interburst Interval Distributions

**FIGURE 4:**
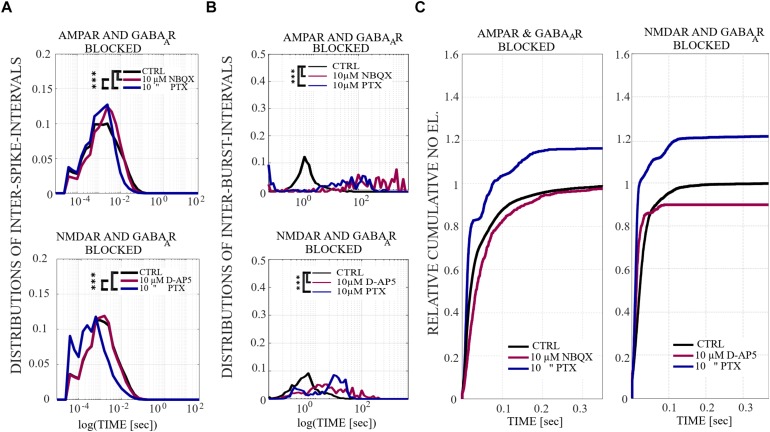
The influence of acute application of the excitatory and inhibitory receptor antagonists on ISIs, IBIs and network-wide electrode recruitment speed at the beginning of the bursts. **(A)**The excitatory and inhibitory receptor dependence on ISIs within NBs. ISI distributions shifted to higher fractions in acutely AMPAR blocked networks (*n* = 7), meaning significantly longer distances between spikes within NBs in neurons probed by 10 μM NBQX (*p*_ranksum_ < 0.001) and, contrariwise, the ISI distribution shifted to lower fractions in disinhibited AMPAR blocked networks (*n* = 7), meaning significantly shorter distances between spikes within NBs in neurons probed by 10 μM PTX (*p*_ranksum_ < 0.001) **(top panel)**. ISI distribution did not differ in an acutely NMDAR blocked network (10 μM D-AP5) (*n* = 6), but disinhibition with 10 μM PTX significantly shifted the ISI distributions toward lower fractions in an NMDAR and GABA_A_R blocked network (*n* = 6), meaning significantly shorter distances between spikes within NBs in neurons without functional NMDARs and GABA_A_Rs (*p*_ranksum_ < 0.001) **(bottom panel)**. **(B)** IBI distributions revealed mainly longer fractions of intervals between NBs in acutely AMPAR blocked condition (10 μM NBQX) (*p*_ranksum_ < 0.001), as well as in acutely AMPAR and GABA_A_R blocked condition (10 μM PTX) (*p*_ranksum_ < 0.001) in comparison to the CTRL condition. Disinhibited networks had shorter IBIs than before disinhibition (*p*_ranksum_ < 0.001) **(top panel)**. IBI distributions shifted toward lower fractions for disinhibited AMPAR-mediated NBs in comparison to the CTRL condition or previous condition (*p*_ranksum_ < 0.001) **(bottom panel)**. Wilcoxon rank sum test and *p*-values were computed for all ISIs and IBIs in each condition and in each culture. If the tests showed similar results and *p*-values for every culture, the results were displayed in **(A,B)**. ^∗∗∗^*p*_ranksum_ < 0.001, ^∗∗^*p*_ranksum_ < 0.01, ^∗^*p*_ranksum_ < 0.05. The x-scale is logarithmic. **(C)** A change in the excitatory and inhibitory receptor balance modulates network-wide electrode recruitment speed at the beginning of the bursts. Network recruitment slowed down by acute AMPAR blockade (10 μM NBQX) and sped up with an increased number of active electrodes by disinhibition (10 μM PTX) in comparison to the CTRL condition **(left panel)**. On contrary, the recruitment of the network sped up with the acute application of NMDAR antagonist (10 μM D-AP5) with less active electrodes and sped up, including more active electrodes in the networks, after acute disinhibition (10 μM PTX) in comparison to the CTRL condition **(right panel)**. The disinhibition of the network by the GABA_A_R antagonist by acute application increased the recruitment speed even more than the NMDAR blockade. Values were normalized to the CTRL conditions. Bin width is 0.1 ms.

The distributions of ISI [s] within NB (i.e., the time difference between the consecutive spikes during NBs) were computed for each condition and for each culture. First the histograms were created using the following values for the edges of the bins: 10^i^, where i is in {−10, −9.8,.…,6}. The mean of ISI distributions from all cultures of the same condition were computed and the values of i were plotted on the x-axis with a logarithmic scale (log(TIME[s]). Statistical analysis was performed for all ISIs between each condition in the same culture using Wilcoxon rank sum test and *p*-values. If the tests showed a similar result for every culture when the same conditions were compared, the differences were considered significant and were displayed as ^∗∗∗^*p*_ranksum_ < 0.001, ^∗∗^*p*_ranksum_ < 0.01, ^∗^*p*_ranksum_ < 0.05 in Figures 4A, 8A and Supplementary Figure S3A.

The distributions of IBIs [s] (i.e., the time difference between the last spike of NB and the first spike of NB + 1) were computed for each condition and for each culture. First, IBI data matrices were smoothed using the Gaussian window method (matlab function *smooth*) with an integer scalar of five for the size of the window and a scalar of 0.65 for SD of the Gaussian window. Then the histograms were created using the following values for the edges of the bins: 10^i^, where i is in {0, 0.05,.…,5}. The mean of IBI distributions from all cultures of the same condition were computed and the values of i were plotted on the x-axis with a logarithmic scale. Statistical analysis was performed for all IBIs between each condition in the same culture using Wilcoxon rank sum test and *p*-values. If the tests showed a similar result for every culture when the same conditions were compared, the differences were considered significant and were displayed as ^∗∗∗^*p*_ranksum_ < 0.001, ^∗∗^*p*_ranksum_ < 0.01, ^∗^*p*_ranksum_ < 0.05 in Figures 4B, 8B and Supplementary Figure S3B.

### Network Recruitment Time Computed as Cumulative Relative Number of Active Electrodes

Cumulative number of electrodes recruited at the onset of a NB, relative to the CTRL, was computed for each condition and each culture. For each NB, the timing of the first spike at each electrode was stored. These stored spike times were used to compute a time vector as follows. We discretized the NB duration, starting at 0.5 s after the onset of NB and using the 0.0001 s discretization time step. For each discrete time step we counted the number of electrodes activated until that time, the obtained cumulative number of electrodes was stored into the time vector. Next, we pooled all the time vectors representing all NBs recorded from the same culture and condition. The mean value of activated electrodes was computed for each time step, by averaging over all NBs. The obtained results illustrate the speed of electrode recruitment, and are shown in Figures 4C, 8C and Supplementary Figure S3C.

### Similarity Analysis of the Spatio-Temporal Patterns

**FIGURE 5:**
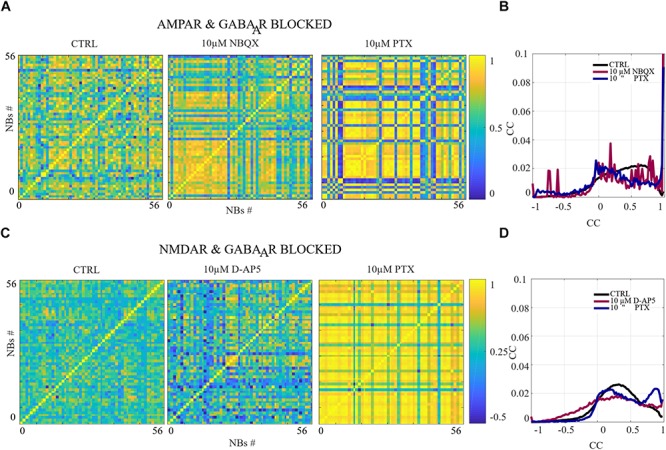
The excitation-inhibition balance changes the spatio-temporal patterns. **(A,C)** CC matrices computed for pairwise-spike-time-difference matrices between each NB in each condition of acute applications. **(B,D)** Mean distributions of CC across the NBs and networks are shown. **(A,B)** The acute AMPAR blockade with 10 μM NBQX changed the distribution of the similarity of spatio-temporal patterns significantly (*p*_ranksum_ < 0.001) in three out of seven networks in comparison to the CTRL condition. However, the number of NBs was too low to evaluate statistical significance in the other four AMPAR blocked networks. The acute disinhibition with 10 μM PTX in AMPAR blocked networks significantly increased (*p*_ranksum_ < 0.001) the similarity of spatio-temporal patterns in four out of seven cultures in comparison to the CTRL condition (*N* = 56 NBs, 7 networks per condition). **(C,D)** The acute NMDAR blockade with 10 μM D-AP5 did not significantly change the CC matrices/diversity of spatio-temporal patterns. The acute GABA_A_R blockade with 10 μM PTX of NMDAR blocked networks significantly increased (*p*_ranksum_ < 0.001) the similarity between spatio-temporal patterns in four out of six cultures in comparison to the CTRL condition (*N* = 56 NBs 6 network per condition). ^∗∗∗^*p*_ranksum_ < 0.001, ^∗∗^*p*_ranksum_ < 0.01, ^∗^*p*_ranksum_ < 0.05.

A more detailed analysis revealed that each NB has a unique structure. The importance of characterizing NB structures has been emphasized in the literature. In Raichman and Ben-Jacob (2008), the NB structure is defined as “momentary spatio-temporal pattern in which neurons at different locations fire spike-trains at different delays relative to each other.” We refer to these unique NB structures as spatio-temporal patterns. To analyze the similarity between each spatio-temporal pattern in different pharmacological conditions, NBs were characterized by the first spike time relative to the burst onset from each electrode, i.e., the rank order of the first spike from each electrode within the NB. Pairwise-spike-time-difference matrices were computed of the difference of the first spike times for each pair of electrodes within each NB. The similarities between NBs were computed as the correlation coefficient (CC) between their pairwise-spike-time-difference matrices. The CC is computed using Matlab implementation of the well-known expression. The CCs, that are close to one indicate similar spatio-temporal patterns between the NBs, CCs close to zero indicate no correlation and CCs close to minus one indicate anticorrelated spatio-temporal patterns. An alternative measure for analysis of spatio-temporal patterns in neuronal cultures has been proposed in the literature (Raichman and Ben-Jacob, 2008). To compare the results of multiple cultures, we computed the distributions of CCs in each condition and in each culture from the CC matrices in Figures 5B,D, 9B,D and Supplementary Figure S4B. Statistical analysis was performed for all CCs between each condition in the same culture using Wilcoxon rank sum test and *p*-values. If the tests showed a similar result for every culture when the same conditions were compared, the differences were considered significant and was displayed as ^∗∗∗^*p*_ranksum_ < 0.001, ^∗∗^*p*_ranksum_ < 0.01, ^∗^*p*_ranksum_ < 0.05 in Figures 5B,D, 9B,D and Supplementary Figure S4B.

## Results

We studied quantitatively the contribution of slow (NMDA) and fast (AMPA) glutamatergic and fast gabaergic (GABA_A_) receptors to the dynamics of spontaneous network activity in cortical cultures *in vitro* (see Figure 1A for illustration). The kinetics of these receptors are known at the single cell and synapse levels, but how their complex interplay affects the network-level activity is not well understood. Our goal was to examine to what extend the fast and slow kinetics of receptor openings play a role in the network activity dynamics. The data studied in this work spontaneously exhibit stereotypical NBs, the periods of intensive activity engaging the majority of neurons and followed by longer silent periods of sparse activity of single spikes. The NBs were characterized by a fast spread of activity across the culture, followed by a slower interval of activity termination. We analyzed the data collected using an *in vitro* experimental setup that simultaneously enabled the precise pharmacological manipulation of synaptic receptor activation and the long-term monitoring of network activity with the MEA technique. We focused on how the excitatory and inhibitory receptors modulate the overall network activity as well as the initiation, maintenance, propagation and termination of NBs. In particular, we assessed how the inhibition mediated by GABA_A_ receptors uniquely shapes the spontaneous NBs guided by AMPA and NMDA receptor transmission.

Our long-term goal is to better understand the complex interplay and mechanisms between the excitatory and inhibitory receptors using both experimental and computational modeling techniques. We contributed to our goal by analyzing a rich set of data recorded under different pharmacological conditions, including the acute and gradual application of each excitatory receptor antagonist and the acute application of inhibitory receptor antagonist to shed light on the network activity dynamics. Four different pharmacological protocols were selected to comprehensively study the contribution of AMPA, NMDA and GABA_A_ receptors to network dynamics (see Table 1). In the first two pharmacological protocols, the immediate acute effects of higher concentrations of NBQX and D-AP5 on spontaneous activity and NBs were studied. One of the ionotropic glutamatergic receptor types was acutely blocked, either AMPARs or NMDARs, and, furthermore, acutely disinhibited the network with GABA_A_ receptor antagonist (see Figure 1B for illustration). In the other two protocols, the network was gradually probed by increasing the concentration of each ionotropic glutamatergic receptor antagonist (see Figure 1C for illustration). The influence of AMPARs on spontaneous network activity dynamics was probed by a NBQX, and the effects of NMDARs were examined by applying D-AP5. We present a quantitative characterization of the complex interplay between NMDAR-, and AMPAR-mediated excitation and GABA_A_R-mediated inhibition. We systematically analyze and compare different measures, including the OFR, the BF, the characteristic NB measures of NB profiles, the ISIs, the IBIs, the recruitment time of active electrodes in the network as well as the similarity of spatio-temporal patterns, computed from multi-unit spike data recorded with MEA under each pharmacological condition.

### Quantifying the Nature of Spontaneous Activity in Cortical Cultures *in vitr*o

Spontaneous network-wide activity developed already at the end of the first week *in vitro* and continued until 20–25 DIV, when the presented experimental recordings were collected. Firstly, the spontaneous activity of CTRL condition consisted of stereotypical NBs that upon initiation rapidly spread across the network with the median of the RP being 88.93 ± 47.10 ms (mean ± std, *n* = 20). Secondly, the observed peak of activity, the MFR in a burst, had median value of 1.90 ± 1.75 Hz (mean ± std, *n* = 20). Thirdly, after reaching the peak, the NBs entered the termination phase which lasted significantly longer, with the median of the FP being 257.24 ± 193.66 ms (mean ± std, *n* = 20). In total, the median of the BL of NBs was 373.17 ± 248.23 ms (mean ± std, *n* = 20), and was followed by relatively long periods of random firing of low intensity with the median of the IBIs being 7.18 ± 4.71 s (mean ± std, *n* = 20). The median number of spikes in NBs (BS) was 204.60 ± 166.22 spikes (mean ± std, *n* = 20) and the median number of active electrodes (RC) in NBs was 26.9 ± 13.52 (mean ± std, *n* = 20) out of the 60 electrodes available in the MEA dishes. Distinct NBs were observed in all the considered experimental conditions. The NBs occurred even in the presence of 30 μM AMPAR (NBQX) or NMDAR (D-AP5) antagonists (see Supplementary Material section). Furthermore, various quantitative measures of NBs significantly changed under different protocols.

### Quantitative Characterization of AMPAR Contribution to Fast Initiation of NBs, Fast Recruitment of Neurons, and Diversity of Spatio-Temporal Patterns

Fast glutamatergic AMPARs are the main mediator of excitatory activity. They play a critical role in the fast initiation of NBs, the fast recruitment of neurons into NBs as well as in the fast activity propagation at the beginning of the NBs. In addition, AMPA and GABA_A_ receptors contributed in decreasing the similarity between spatio-temporal patterns. These functions of AMPARs were studied using two complementary experimental protocols, the acute and gradual blocking of AMPARs. Moreover, we showed differences between the acute and gradual protocols. The gradual application of AMPAR blockade induced SBs, which were not observed in acutely AMPAR blocked networks. We compare the major differences between the gradual and acute applications in the last paragraph of the section “Results.” In what follows, we present the data obtained using acute protocol quantified using a versatile set of activity measures (see section “Materials and Methods”).

The network dynamics were probed by applying acutely high concentrations of 10 μM (the results obtained when applying 30 μM of NBQX are shown in Supplementary Material section) of NBQX to explore the immediate acute effects of AMPAR antagonists on network dynamics. The results were consistent with both concentrations. Therefore, we show here only the results with 10 μM NBQX and in the Supplementary Material section the results with 30 μM; the raster plots of network activity demonstrate characteristic long bursts (Figures 2A, 3A, middle subpanels) with significantly longer IBI (*p*_ranksum_ < 0.001; Figure 4B, top panel) in comparison to the CTRL condition. The OFR (*p*_ranksum_ < 0.01) and BF (*p*_ranksum_ < 0.001) significantly decreased with acute NBQX application (Figure 2B) when compared to CTRL. The BF decreased more than the OFR (Figure 2B), meaning that NBs occurred less frequently compared to CTRL. The burst measure analysis showed that BL, FP and BS significantly increased (*p*_ranksum_ < 0.001) in comparison to the CTRL condition (Figure 3B). The fraction of ISI distributions significantly increased (*p*_ranksum_ < 0.001), meaning longer ISIs with complete AMPAR blockade (Figure 4A, top panel). This confirms that AMPARs are needed for fast spiking within the NBs. The IBI distributions shifted toward higher values, meaning significantly less frequent NBs than in the CTRL condition (*p*_ranksum_ < 0.001) (Figure 4B, top panel). This observation shows that AMPARs have an important role in the initiation of NBs. The network-wide electrode recruitment time increased also after acute AMPAR blockade, meaning slower network recruitment (Figure 4C, left panel). The acute AMPAR blockade significantly (*p*_ranksum_ < 0.001) increased the similarity of spatio-temporal patterns in three out of seven networks in comparison to the CTRL condition (Figures 5A,B). However, the number of NBs was too low to evaluate statistical tests for the other four acutely AMPAR blocked networks.

### Quantitative Characterization of NMDAR Contribution to Spatial and Temporal Maintenance of the Propagating Network Bursts

The slower glutamate receptors, the NMDARs, contributed to the overall network excitability via manipulation of spatio-temporal patterns of network activity. In the temporal domain, their contribution maintained the network activity particularly during the termination phase of the NBs. In the spatial domain, the blocking of NMDARs reduced the number of active electrodes. NMDAR blockade reduced also the time needed for activity to recruit all the electrodes, indicating that NMDARs or another mechanism influencing NMDARs, e.g., the inhibition, slowed down the activity recruitment in these networks.

The role of NMDARs on spontaneous NB dynamics were studied probing the culture by acute application of 10 μM of NMDAR antagonist D-AP5. The consistent findings were obtained when probing the cultures with a higher concentration of 30 μM D-AP5, these results are presented in the Supplementary Material section. The raster plots of network activity demonstrate characteristic short NBs (Figure 2C, middle subpanels). The results confirmed that the OFR significantly decreased (*p*_ranksum_ < 0.01) and BF also slightly decreased when acutely blocking the NMDARs (Figure 2D). The OFR decreased more in the NMDARs blocked networks than in the AMPARs blocked networks in comparison to the CTRL condition in all acute applications (Figures 2B, left panel, D, left panel). On the contrary, the BF decreased more in AMPAR blocked networks than in NMDAR blocked networks in comparison to the CTRL condition (Figures 2B, right panel, D, right panel), indicating that NMDARs do not contribute as much to the BF as AMPARs. The BL (*p*_ranksum_ < 0.01), FP (*p*_ranksum_ < 0.01), RP (*p*_ranksum_ < 0.05), BS (*p*_ranksum_ < 0.01) and RC (*p*_ranksum_ < 0.05) significantly decreased when acute 10 μM D-AP5 was applied (Figures 3C,D). This means that NMDARs are responsible for maintaining NBs in these networks. Intriguingly, the blockade of NMDARs reduced the RC in all applications, which suggests that the NMDARs have an important spatial role in overall NB activity. The ISI distributions were not modulated by acute NMDAR blockade (Figure 4A, bottom panel). On the contrary, the IBI distributions shifted toward higher fractions, indicating significantly longer (*p*_ranksum_ < 0.001) intervals between NBs in the NMDAR blocked condition than in the CTRL condition (Figure 4B, bottom panel). Moreover, in the NMDAR blocked condition the IBIs intervals were shorter than in the AMPAR blocked condition (Figure 4B) which stressed the dependence of AMPARs for the initiation of NBs. The recruitment time shortened when the NMDARs were antagonized (Figure 4C, right panel), meaning that either NMDARs or, e.g., the interplay between NMDARs and inhibition slow down the activity propagation. The results of the similarity analysis of the spatio-temporal patterns indicate that the acute NMDAR blockade did not significantly change the similarity between spatio-temporal patterns, indicating that AMPARs facilitate the diversity (*p*_ranksum_ < 0.001) of the activity propagation and that NMDARs do not diversify the spatio-temporal patterns (Figures 5C,D).

### GABA_A_Rs Dampen the Termination Phase of Network Bursts and Decrease the Burst Frequency in the NMDAR-Mediated Networks

So far, we described the changes in network-wide dynamics when blocking individual excitatory receptors through acute application of a receptor antagonist. In what follows, we examine the combined contributions of pairs of receptors. This is done first by acute blocking of one of the considered glutamatergic receptors described above and then by acute disinhibition. In this section we present the quantitative characterization of a combined contribution of AMPAR and GABA_A_R blockade to the network activity. The ionotropic GABA_A_Rs play a critical role in the fast inhibition of neurons. In the presence of NMDARs, GABA_A_Rs contributed particularly to the inhibition of the termination phase of NBs as well as to NB frequency in NMDAR-mediated networks. Finally, the overall spiking frequency in NMDAR-mediated networks was not as strongly inhibited by GABA_A_Rs as the spiking frequency in AMPAR-mediated networks.

To assess how GABA_A_ receptors shape the NMDAR-mediated spontaneous NB dynamics, first the AMPARs were acutely blocked (10 μM NBQX, i.e., NMDAR-mediated networks), and then the networks were acutely disinhibited by applying 10 μM PTX. Disinhibition did not change the OFR, but it significantly increased (*p* < 0.05, *n* = 7) the BF in comparison to the solely AMPAR blocked condition (Figure 2B). However, the BF remained significantly lower after disinhibition when compared to the CTRL condition (*p* < 0.001, *n* = 7) (Figure 2B, right panel), meaning that GABA_A_Rs inhibited the NMDAR-mediated NB frequency. Disinhibited NMDAR-mediated (AMPAR and GABA_A_R blocked) burst profiles were truncated in comparison to long NMDAR-mediated profiles (Figure 3A), meaning that GABA_A_Rs slow down the termination phase of the NMDAR-mediated NBs. A detailed burst measure analysis showed that the BL (*p* < 0.001, *n* = 7), FP (*p* < 0.001 *n* = 7) and BS (*p* < 0.05, *n* = 7) significantly decreased by disinhibition in comparison to the previous AMPAR blocked condition (Figure 3B). However, the BL (*p* < 0.01 *n* = 7), FP (*p* < 0.05, *n* = 7) and BS (*p* < 0.05, *n* = 7) significantly increased in comparison to the CTRL condition (Figure 3B). The ISI distribution shifted significantly (*p* < 0.001, *n* = 7) toward lower values in NMDAR-mediated networks by acute disinhibition when compared to the CTRL condition or the solely AMPAR blocked conditions, meaning that GABA_A_Rs inhibit NMDAR-mediated spiking (Figure 4A, top panel). However, the shift in ISI distributions was not as profound as for the ISI distributions in networks with dominantly AMPAR-mediated synaptic transmission (Figure 4A). The IBI distributions were significantly higher (*p* < 0.001) in disinhibited AMPAR blocked networks when compared to the CTRL condition (Figure 4B, top panel). IBIs significantly decreased (*p* < 0.01) in four out of seven cultures and did not change in three out of seven cultures when compared to the previous AMPAR blocked condition. This indicates that disinhibition shortens IBIs in NMDAR-mediated networks (Figure 4B, top panel). The network recruitment time decreased in all cultures (*n* = 7) (Figure 4C, left panel), indicating that GABA_A_Rs contribute by inhibiting the network recruitment at the beginning of the NMDAR-mediated NBs as well. The acute disinhibition of NMDAR-mediated networks significantly increased (*p*_ranksum_ < 0.001) the similarity of spatio-temporal patterns in four out of seven cultures (three cultures contained too few NBs to reliably compute statistical significance) (Figures 5A, right panel, B).

### GABA_A_Rs Contribute to Inhibiting the Spiking Frequency and Preventing the Fast Spread of Activity Propagation in the AMPAR-Mediated Networks

In this section, we present the quantitative characterization of a combined contribution of NMDAR and GABA_A_R blockade to the network activity. The blocking of GABA_A_Rs significantly increased the number of active electrodes in AMPAR-mediated networks. GABA_A_Rs dampened the initiation phase of the NB activity and slowed down the network recruitment in AMPAR-mediated networks. In the presence of AMPARs, GABA_A_Rs contributed particularly to the inhibition of spiking frequency. GABA_A_Rs balanced the AMPAR-mediated activity to maintain the dynamics of activity, suggesting crucial interplay between fast GABA_A_Rs-mediated inhibition and fast AMPAR-mediated spiking.

In order to study the influence of GABA_A_ receptors on shaping the AMPAR-mediated NB activity dynamics we first acutely blocked NMDARs using 10 μM D-AP5, and then acutely disinhibited the networks by applying 10 μM of PTX. The results showed that the disinhibition significantly increased (*p* < 0.01, *n* = 6) the OFR, but not BF in comparison to the NMDAR blocked condition (Figure 2D). The BL (*p* < 0.01, *n* = 6), FP (*p* < 0.01, *n* = 6), MFR (*p* < 0.01, *n* = 6), BS (*p* < 0.01, *n* = 6) and RC (*p* < 0.05, *n* = 6) significantly increased in comparison to the previous NMDAR blocked condition, meaning that GABA_A_Rs strongly inhibited the spiking frequency within the NBs (Figure 3D). In addition, disinhibition increased the spatial spread of extracellular activity in AMPAR-mediated networks, meaning that GABA_A_Rs effectively inhibited the AMPAR-mediated activity across the cultures (Figure 3D, RC). Contrary to the NMDAR-mediated networks, in AMPAR-mediated networks the distributions of ISIs did not significantly deviate from the one recorded under the CTRL condition (Figure 4A). Interestingly, disinhibition significantly reduced (*p* < 0.001) the ISIs when compared to both the CTRL and the AMPAR-mediated condition (Figure 4A, bottom panel). Although, in NMDAR-mediated networks, the mean of the ISI distribution also shifted toward a lower value after disinhibition, the change was not as significant as in the AMPAR-mediated networks (Figure 4A). This means that GABA_A_Rs strongly inhibited the spiking frequency in AMPAR-mediated networks and suggest important interplay between the GABA_A_Rs-mediated inhibition and the AMPAR-mediated fast spiking. The IBIs were significantly increased (*p* < 0.001, *n* = 6) in disinhibited AMPAR-mediated networks in comparison to the CTRL condition and the condition before disinhibition (Figure 4B, bottom panel). The IBIs were significantly reduced (*p* < 0.001) by disinhibition in half of the networks and significantly increased (*p* < 0.01) in the other half in comparison to the solely AMPAR-mediated condition (notice the bidmodal distribution in Figure 4B, bottom panel). Disinhibition decreased the recruitment time of electrodes in all cultures (*n* = 6) compared to the CTRL condition and the AMPAR-mediated condition (Figure 4C, right panel). This means that disinhibition significantly accelerated the recruitment of neurons in these networks while GABA_A_Rs tended to maintain the steady speed of recruitment. The acute disinhibition of AMPAR-mediated networks significantly increased (*p*_ranksum_ < 0.001) the similarity of spatio-temporal patterns in four out of six cultures (Figures 5C,D).

### Gradual AMPAR Blockade Induced Super Bursts That Share Similar Spatio-Temporal Patterns

**FIGURE 6:**
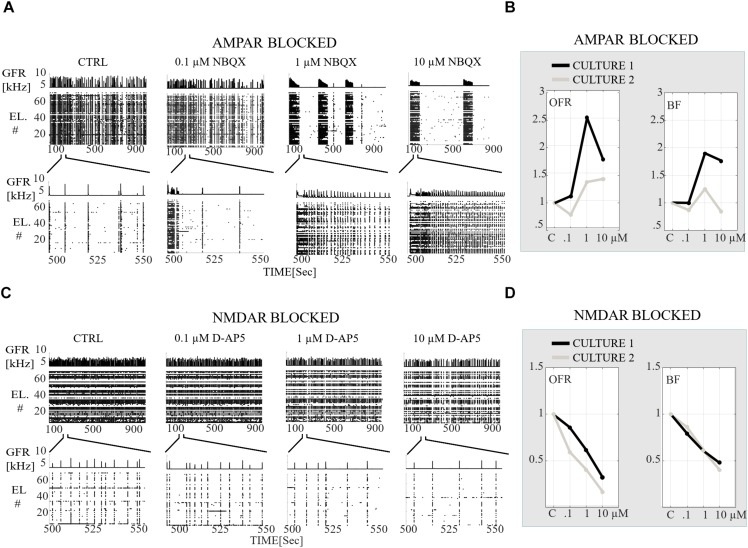
Receptor-dependent network-wide activity probed by gradual application of AMPA and NMDA receptor antagonists, relative changes in OFR [Hz] and in BF [NB/min]. **(A)** Raster plots of network activity of the CTRL recording probed by gradual application of AMPAR antagonist (0.1, 1, and 10 μM NBQX). In each subpanel, the global firing rates (GFR [kHz]) are displayed on top of the raster plots of spike times [s] from electrodes (EL #) in each condition. Top row shows the completely analyzed recording of 900 s, and the bottom row enlargements of the 60 s to display fine details of the activity. **(B)** Relative change in OFR and BF with respect to those obtained from the CTRL condition as shown for each recording (*n* = 2 cultures). This relative change is computed as described in the section “Materials and Methods”. The values of the applied NBQX concentrations are shown on the x-axis. **(C)** Same as **(A)** except that a CTRL recording is probed by gradual application of NMDARs antagonists (0.1, 1, and 10 μM D-AP5). **(D)** Same as **(B)** except that the CTRL recordings are compared to condition when NMDARs are gradually antagonized with 0.1, 1, and 10 μM D-AP5. Gradual AMPAR blockade increased the OFR in both cultures in comparison to the CTRL condition **(B)**. Gradual application of NMDAR antagonist gradually decreased the OFR and BF in comparison to the CTRL condition **(D)**.

**FIGURE 7:**
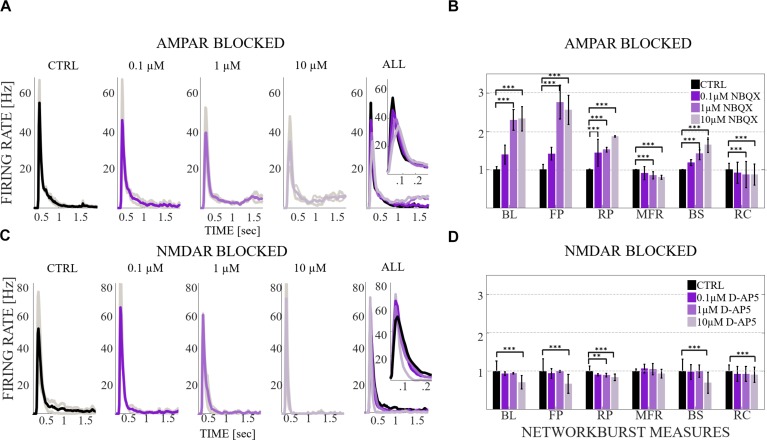
The influence of gradual application of the excitatory receptor antagonists on NB profiles and burst measures. **(A)** The burst profiles [Hz] from two congruent experiments where the data of the CTRL recording (1^st^ subpanel) is compared to the data of the recording conditions when AMPARs (2^nd^, 3^rd^, and 4^th^ subpanels) are antagonized with 0.1, 1, and 10 μM NBQX. Thin gray lines represent the average NB profiles of each culture in each condition, whereas the thick line represents the average over both cultures. The profiles are aligned with each other at the time point when the MFR is reached. The presented profiles are computed using the spike-data from Figure 6, processed as described in section “Materials and Methods”. The 1^st^, 2^nd^, 3^rd^, and 4^th^ subpanels show the first 1.5 s of burst profiles. The 5^th^ subpanel shows all four average NB profiles in the same panel and enlargement of the first 300 ms of all four profiles to display the fine details at the beginning of the NBs. Bin width is 0.01 s. **(B)** The relative change of characteristic burst measures extracted from burst profiles, including BL, FP, RP, MFR, BS and RC (see section “Materials and Methods”). Each bar represents the mean of the medians ± SD divided by mean, i.e., coefficient of variation from both cultures. The top panel shows the relative change in networks probed by a gradual blockade of AMPARs (0.1, 1, and 10 μM NBQX). **(C)** Same as **(A)** except that the data of the CTRL recording (1^st^ subpanel) is compared to the data of the recording conditions when NMDARs (2^nd^, 3^rd^, and 4^th^ subpanels) are antagonized with 0.1, 1, and 10 μM D-AP5. **(D)** Same as **(B)** except that the relative change in networks is probed by a gradual blockade of NMDARs (0.1, 1, and 10 μM D-AP5). Wilcoxon rank sum test and *p*-values were computed for all burst measures in each condition and in each culture. If the tests showed a similar result for both cultures, the significance was displayed in **(B,D)**. BL, FP, RP and BS gradually increased by a gradual AMPAR blockade **(B)** and gradually decreased by a gradual NMDAR blockade **(D)**. MFR gradually decreased by AMPAR blockade **(B)**. RC decreased by both AMPAR and NMDAR blockades **(B,D)**. ^∗∗∗^*p*_ranksum_ < 0.001, ^∗∗^*p*_ranksum_ < 0.01, ^∗^*p*_ranksum_ < 0.05.

**FIGURE 8:**
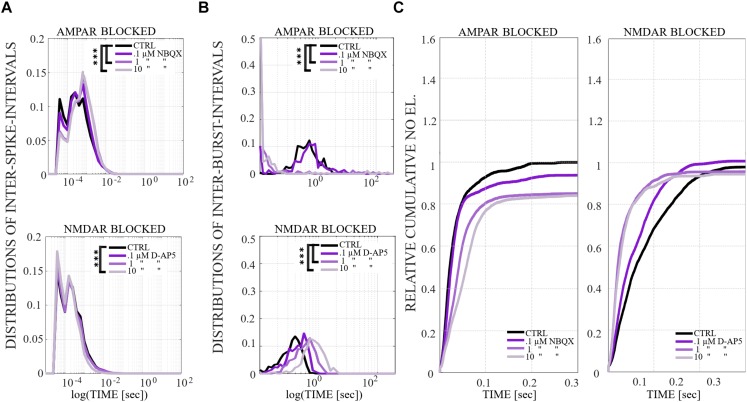
The influence of gradual application of the excitatory receptor antagonists on ISIs, IBIs and network-wide electrode recruitment speed at the beginning of the bursts. **(A)** The excitatory receptor dependence on ISIs within NBs. Average ISI distribution gradually shifts toward higher fractions in gradually AMPAR blocked networks (*n* = 2) (top panel), meaning significantly longer distances between spikes within NBs in neurons probed by 1 and 10 μM NBQX (*p*_ranksum_ < 0.001). In contrast, the average ISI distribution shifts toward slightly lower fractions in gradually NMDAR blocked networks (*n* = 2) (bottom panel), meaning significantly shorter distances between spikes within NBs in neurons probed by 10 μM D-AP5 (*p*_ranksum_ < 0.001). **(B)** A change in the excitatory receptor balance modulates the duration of IBIs and thus the frequency of NB events. Neurons in gradually AMPAR blocked networks with 1 and 10 μM NBQX expressed bimodal distributions with significantly shorter and longer IBIs than neurons in CTRL or in 0.1 μM NBQX blocked networks (*p*_ranksum_ < 0.001) (top panel). The IBI distributions gradually shifted toward higher fractions in gradually NMDAR blocked networks (bottom panel), meaning significantly longer distances between NBs in neurons probed by 1 and 10 μM D-AP5 in comparison to the CTRL condition (*p*_ranksum_ < 0.001). Wilcoxon rank sum test and *p*-values were computed for all ISIs and IBIs in each condition and in each culture. If the tests showed similar results and *p*-values for every culture, the results were displayed in **(A,B)**. ^∗∗∗^*p*_ranksum_ < 0.001, ^∗∗^*p*_ranksum_ < 0.01, ^∗^*p*_ranksum_ < 0.05. The x-scale is logarithmic. **(C)** A change in the excitatory receptor modulates network-wide electrode recruitment speed at the beginning of the NBs (left panel). The network recruitment speed slowed down gradually with gradual AMPAR blockade (0.1, 1, and 10 μM NBQX) (left panel) and sped up gradually with gradual NMDAR blockade (0.1, 1, and 10 μM D-AP5) (right panel). Values were normalized to the CTRL conditions. Bin width is 0.1 ms.

In order to gradually study the influence of AMPARs on spontaneous network dynamics, the networks were also gradually blocked in a concentration-dependent manner using 0.1, 1, and 10 μM of NBQX (the total added NBQX concentration was 11.1 μM). Intriguingly, these networks exhibited a very strong occurrence of SBs (see section “Materials and Methods”) when using 1 and 10 μM of NBQX (*n* = 2) (Figure 6A). The SBs lasted ∼100 s and were separated by longer silent periods. The duration of these silent periods was dependent on the concentration of applied antagonist, i.e., when applying 1 μM of NBQX the duration was ∼3 min, and when applying 10 μM of NBQX it was ∼6 min (Figure 6A). The gradual application of the AMPAR antagonist increased the OFR when applying 1 and 10 μM of NBQX and increased the BF when applying 1 μM of NBQX in comparison to the CTRL condition (*n* = 2) (Figure 6B). At the end of the gradual application protocol (11,1 μM NBQX), the NBs lasted longer with the median BL being 305.75 ± 80.26 ms (mean ± std, *n* = 2) in comparison to the CTRL condition with the median of the BL being 137.25 ± 9.56 ms (mean ± std, *n* = 2) (Figure 7B). Additionally, the intervals between NBs became shorter with the median of IBIs being 645.86 ± 302.00 ms (mean ± std, *n* = 2) in comparison to the CTRL median value of 5916 ± 686.00 ms (mean ± std, *n* = 2) (Figure 8B, top panel).

**FIGURE 9:**
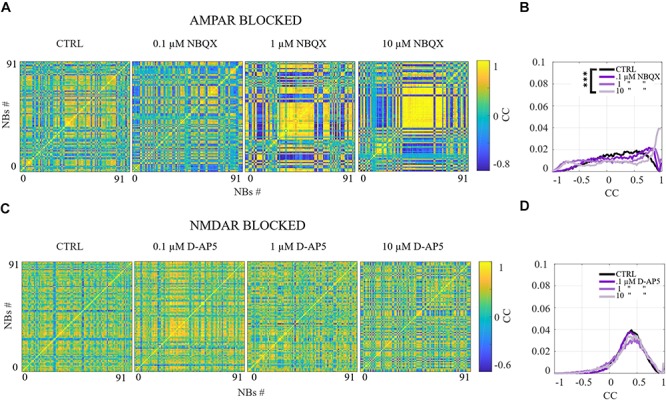
The gradual AMPA receptor blockade changes the spatio-temporal patterns which is not seen in the gradual NMDA receptor blockade. **(A,C)** CC matrices computed for pairwise-spike-time-difference matrices between each NB in each condition of gradual applications. **(B,D)** Mean distributions of CC across the NBs and networks are shown. **(A)** The spatio-temporal patterns became less diverse with gradual 10 μM AMPAR blockade (*N* = 91 NBs). **(B)** The distribution of CC in AMPAR blocked condition (10 μM NBQX) obtained an increased tail around one in addition to another lower peak around minus one, this indicates polarization among the NBs with respect to spatio-temporal patterns of activity in cultures treated with gradual AMPAR blockade (*N* = 91 NBs). **(C,D)** The gradual NMDAR blockade did not have an effect on the similarity of spatio-temporal patterns in comparison to the CTRL condition (*N* = 91 NBs). The gradual AMPAR blockade with 10 μM NBQX significantly decreased (*p*_ranksum_ < 0.001) the diversity of spatio-temporal patterns in comparison to the CTRL condition **(A,B)**. The gradual NMDAR blockade did not significantly change the diversity of spatio-temporal patterns **(C,D)**. ^∗∗∗^*p*_ranksum_ < 0.001, ^∗∗^*p*_ranksum_ < 0.01, ^∗^*p*_ranksum_ < 0.05.

A more detailed analysis of NB measures showed that the increase in OFR results from the significant increase in BL, FP, RP and BS (*p*_ranksum_ < 0.001) (Figure 7B). MFRs and RC significantly decreased (Figure 7B) and the MFR was reached later by concentration-dependent manner (Figure 7A, the inset). The median ISI significantly increased when using 1 and 10 μM of NBQX (*p*_ranksum_ < 0.001) (Figure 8A, top panel), indicating longer distances between spikes within NBs. In experiments where the gradual blocking of AMPARs was performed, the long SBs disintegrated into a number of shorter NBs, separated by visible but shorter IBIs. The median IBIs were significantly shorter (*p*_ranksum_ < 0.001) in comparison to the CTRL condition (Figure 8B, top panel). The recruitment time of neurons in a network increased when gradually applying the AMPAR antagonist (Figure 8C, left panel), meaning that the fast recruitment of neurons attending to NBs is dependent on AMPARs. The results of the similarity analysis of the spatio-temporal patterns indicate that the gradual AMPAR blockade significantly increased the similarity between spatio-temporal patterns, meaning that AMPARs support the diversity (*p*_ranksum_ < 0.001) of the activity propagation (Figures 9A,B).

The role of NMDARs on spontaneous network dynamics was also assessed with the gradual blockade of NMDARs in a concentration-dependent manner using 0.1, 1, and 10 μM D-AP5 (the total added D-AP5 concentration was 11.1 μM). The results showed that the OFR and BF decreased when applying an increased concentration of the excitatory antagonist (D-AP5) (Figures 6C,D). A more detailed analysis of burst measures showed that the BL, FP, RP, BS and RC significantly decreased (*p*_ranksum_ < 0.001) when applying 10 μM D-AP5 (Figure 7D). The comparison of burst profiles and zooming into the first 200 ms showed that the BL, in particular the FP of the NBs, diminished when using 10 μM D-AP5 (Figure 7C, the inset). IBIs significantly increased (*p*_ranksum_ < 0.001) with 1, and 10 μM D-AP5 (Figure 8B, bottom panel). In addition, our results showed that these networks were recruiting neurons faster to the NBs than the CTRL networks, meaning that NMDARs or related mechanisms slow down the recruitment of neurons at the beginning of NBs (Figure 8C, right panel). Finally, we show that there are no changes in the similarity measures of the spatio-temporal patterns in NMDAR-blocked cultures, indicating that the NMDARs do not diversify spatio-temporal patterns of NBs (Figures 9C,D). However, we show that NMDARs contribute characteristic SBs composed of tens of slow NBs with highly repetitive spatio-temporal patterns in gradually AMPAR blocked networks (Figures 9A,B).

In what follows, we compare the major differences between the results of gradual and acute blockades. Intriguingly, the gradual application of an AMPAR blockade induced SBs (Figure 6A), which were not observed in acutely AMPAR blocked networks (Figure 2A, middle panel). Although there were long silent periods between the SBs, the OFR significantly increased in comparison to the CTRL condition in the case of gradual AMPAR blockade (Figures 6A,B). In contrast, the OFR significantly decreased in comparison to CTRL in the case of acute AMPAR blockade (Figure 2B, left panel). In the case of acute AMPAR blockade also the BF significantly decreased when compared to the CTRL condition (Figure 2B, right panel), which we did not observe in the networks probed by a gradual AMPAR blockade (Figure 6B, right panel). However, the BL, FP, BS and ISIs increased in both experimental protocols (Figures 3B, 7B, 4A, top panel, 8A, top panel). Furthermore, the IBIs decreased in networks with a gradual AMPAR blockade (Figure 8B, top panel), but increased in networks with an acute AMPAR blockade (Figure 4B, top panel) in comparison to the CTRL condition. The recruitment time increased similarly in both gradual and acute applications of the AMPAR blockade (Figures 4C, left panel, 8C, left panel). The spatio-temporal patterns became similar when gradually blocking the AMPARs (Figures 9A,B). However, the acute blocking of AMPARs gave rather small number of NBs. Thus for this experimental condition we could not to evaluate the similarity of NBs with sufficient statistical significance (Figures 4A,B). Finally, there were no differences when gradually and acutely blocking the NMDARs nor the GABA_A_Rs.

## Discussion

We systematically analyzed the unique features of NBs, including the initiation, maintenance, propagation and termination of NBs, which emerge from a complex interplay between fast AMPA and slow NMDA glutamatergic and fast GABA_A_ gabaergic receptors when embedded into a self-organized network. We analyzed the data collected from P0 rat cortical neurons between 20–25 DIV using the MEA recording technique with receptor antagonists. The kinetics of the receptors are known at the single cell and synapse levels, but their contribution to NBs is not well understood. We show how the spontaneous NBs are modified by the distinctive kinetics of each of the ionotropic excitatory and inhibitory receptors in these networks. In particular, (1) AMPARs contributed to the fast initiation of NBs, the fast recruitment of network activity and more versatile spatio-temporal patterns of network activity; (2) NMDARs maintained the NBs temporally and spatially as well as slowed down the spread of activity propagation; (3) GABA_A_Rs inhibited the spiking frequency and prevented the fast spread of activity propagation in the AMPAR-mediated networks; (4) GABA_A_Rs further dampened the termination phase of the NBs and the NB frequency in the NMDAR-mediated networks; in addition, (5) the NMDAR- and GABA_A_R-mediated networks exhibited characteristic SBs composed of slow NBs with highly repetitive spatio-temporal patterns in the presence of a gradual AMPAR blockade. To the best of our knowledge, all these unique features of the NBs emerging from the interplay between excitatory and inhibitory receptors have not been previously reported using the MEA recording technique, pharmacological protocols and rat P0 cortical cell cultures referred to herein.

Dissociated *in vitro* cell cultures have been previously used as model systems to study cortical network activity with MEA technique. The MEA technique is a widely used, reliable and feasible recording technique for high-throughput screening of network dynamics, particularly when multiple cell cultures and experimental protocols are considered similarly to this study. The network activity has been studied in cell cultures obtained from the neocortex of P0 (Shahaf and Marom, 2001; Eytan and Marom, 2006; Teppola et al., 2011; Weihberger et al., 2013; Reinartz et al., 2014; Haroush and Marom, 2015; Okujeni et al., 2017) and E17-18 (Robinson et al., 1993; Maeda et al., 1995; Kamioka et al., 1996; Jimbo et al., 2000; Opitz et al., 2002; van Pelt et al., 2004; Chiappalone et al., 2006; Wagenaar et al., 2006; Baltz et al., 2010; Fong et al., 2015) rats. In addition, network activity has been studied in cultures prepared from other areas of the rodent central nervous system, including hippocampus (Arnold et al., 2005; Mazzoni et al., 2007; Chen and Dzakpasu, 2010; Niedringhaus et al., 2013; Eisenman et al., 2015; Slomowitz et al., 2015; Suresh et al., 2016; Lonardoni et al., 2017) and spinal cord (Keefer et al., 2001; Gramowski et al., 2004; Legrand et al., 2004; Ham et al., 2008). In this study, we analyzed the activity recorded from rat P0 networks under a rich set of pharmacological protocols. In these protocols excitatory (AMPA, NMDA) and inhibitory (GABA_A_) receptors were systematically blocked, and consequently the excitation-inhibition balance was modified. The impact of changes in excitation-inhibition balance to NB dynamics was addressed. We used an extensive set of data analysis methods to quantitatively explain the observed mechanisms. This approach allowed us to not only closely inspect the data and the studied mechanisms but also to present the data in a format that facilitates the computational modeling of the system.

The adopted *in vitro* experimental setup enables the precise manipulation of pharmacological conditions in the extracellular environment while recording the spatio-temporal evolution of network activity with the MEA technique over many hours. The MEA technique allows the robust monitoring of the uniformly sampled extracellular network activity for several hours (Egert et al., 2002). Precise pharmacological manipulations combined with long-term activity recordings are difficult *in vivo* (Yang et al., 2016). In turn, the main drawbacks of *in vitro* models are the lack of architecture and sensory inputs from other brain areas, which lead to the formation of the network without natural function after development. Although this reduced approach yields differences in observations between network properties *in vitro* and *in vivo*, general phenomena, such as cell homeostasis and synaptic transmission, have been found both *in vitro* and *in vivo*. In addition, *in vitro* cell cultures are conventionally used in pharmacological testing and design. To address the central question of this study, the quantitative analysis of NB features emerging from the complex interplay of excitatory and inhibitory receptors, *in vitro* cultures combined with the MEA recording technique provide an optimal setup.

The same experimental setup has been used to demonstrate a number of properties of spontaneous and evoked activity. Previous studies show that the network activity appears in the form of uncorrelated firing of spikes at the end of the first week *in vitro* (Shahaf and Marom, 2001; Marom and Shahaf, 2002). Consistent with previous research (Linne et al., 1996), we also observed uncorrelated spikes in networks used in this study during the first week *in vitro* (data not shown). At the 2^nd^ week *in vitro*, the stereotypical NBs became prominent in the network activity and persisted throughout the lifetime of the cultures (data not shown) similarly to previous studies (Maeda et al., 1995; Kamioka et al., 1996; Nakanishi and Kukita, 1998; Marom and Shahaf, 2002; Wagenaar et al., 2006; Baltz et al., 2010). During the 3^rd^ week *in vitro*, we observed that the NBs were more variable and the NB frequency had increased in comparison to previous weeks similar to others (Marom and Shahaf, 2002; Wagenaar et al., 2006; Baltz et al., 2010). The overall level of activity as well as the structure and frequency of NB properties have been shown to depend on the used animal species, brain location, age of the donor rats (Lin et al., 2002), seeding density (Wagenaar et al., 2006) and the age of the culture (Wagenaar et al., 2006). Denser cultures are shown to exhibit bursting more actively and earlier than sparser cultures (Wagenaar et al., 2006). According to the definition from Wagenaar et al. (2006), we used data from cultures that provide dense (2000 cells/mm^2^) and mature (20–25 DIV) networks in order to provide as stable and comparable recording conditions as possible for every culture (Wagenaar et al., 2006). These recording conditions allowed us to systematically and quantitatively characterize the measures extracted from each NB. Finally, our control networks showed a steady occurrence of network bursting (NB) with frequency of 0.120 ± 0.079 Hz (mean ± std, *n* = 20) with the results being consistent with previous studies of similar neocortical postnatal preparations (Weihberger et al., 2013; Okujeni et al., 2017), see also (Teppola et al., 2011). Next, we discuss the unique features of the network activity that emerged from the contributions of the main mediators of synaptic transmission in rat postnatal cortical cultures *in vitro*.

We first assessed the contribution of fast glutamatergic AMPARs. These receptors play a critical role in the initiation of NBs and in the fast recruitment of neurons into the NBs. In the section “Results,” we carefully evaluated these contributions using a number of suitable quantitative measures. Our results are consistent with previous studies which showed the critical contribution of AMPARs to the early phase (∼0–25 ms) of NBs in cortical cell cultures *in vitro* (Jimbo et al., 2000; Wagenaar et al., 2004) as well as with recent studies which demonstrated that the NMDAR blockade [with 50 μM APV/20 μM CPP ((±)-3-(2-Carboxypiperazin-4-yl)propyl-1-phosphonic acid)] significantly reduced the duration of spontaneous NBs compared to the control condition in embryonic cortical and hippocampal cultures (Fong et al., 2015; Suresh et al., 2016). In organotypic neonatal visual cortex explants network activity has been shown to be strongly suppressed after applying a selective NMDA receptor blocker (20 μM APV) and began again to rise after 30 min or more exposure in 1- and 2-weeks old explants, whereas the activity levels did not rise after initial suppression in 3-weeks old explants (Corner et al., 2002). Similarly to Corner et al., 2002, we did not observe any rise in the network activity during the 1 h recording period after acute application of the NMDA receptor blocker D-AP5 in the 3 week-old networks. Furthermore, we showed that the interplay between AMPARs and GABA_A_Rs significantly increases the diversity of spatio-temporal patterns. This important contribution has not been shown before in *in vitro* preparations, to the best of our knowledge. Jointly, the presented results suggest the complex contribution of fast AMPARs and fast GABA_A_Rs in the spatiotemporal organization of network activity, rather than only exciting or reducing the overall level of activity.

Next, we addressed the contribution of NMDARs to the network activity. We showed that the NBs persist even in the presence of a high concentration of AMPAR antagonists, such as 30 μM of NBQX. Further analysis of the recorded NBs showed that NMDARs maintain the elevated network activity particularly during the late phase of the NBs. Together with GABA_A_Rs, the NMDARs contribute to slowing down the recruitment of neurons into the NBs. A body of literature supports our findings by showing that AMPAR blocking (by 40/50/100 μM of CNQX) only partially reduced the FR in cortical cultures taken from E18 rats (Ramakers et al., 1993; Martinoia et al., 2005; Fong et al., 2015). This reduction was primarily obtained through reduction in the NB frequency while the level of spiking during NBs remained the same or even increased compared to the control condition (Ramakers et al., 1993; Martinoia et al., 2005; Fong et al., 2015). In addition, Jimbo et al. (2000) showed that in evoked NBs the slower termination phase lasted approximately 25–300 ms (Jimbo et al., 2000). All of these studies showed that the application of an AMPAR antagonist increases the NB duration while the application of an NMDAR antagonist reduces the termination phase (Ramakers et al., 1993; Jimbo et al., 2000; Martinoia et al., 2005; Fong et al., 2015). These findings support our results. Previous studies using dissociated hippocampal cultures taken from E18 rats showed complete abolishing of NBs in the presence of a selective AMPAR antagonist CNQX already at the concentration of 20 μM, different to our findings (Suresh et al., 2016; Lonardoni et al., 2017). The deviations in results can emerge from differences in the data-analysis methods applied to data collected using high-density MEA in Mazzoni et al. (2007) (as opposed to the standard 59 TiN electrode MEA used here), from a relatively short recording period (15 min, compared to our 50 min long recordings) or from the embryonic origin of these cell cultures (Suresh et al., 2016; Lonardoni et al., 2017). It has been demonstrated that the use of higher electrode density may affect the statistics of network activity measures in dissociated cultures (Gandolfo et al., 2010; Maccione et al., 2010; Lonardoni et al., 2015). Furthermore, the age of the donor animal affects the properties of the excitatory synapses in cultured neurons (Lin et al., 2002). More precisely, Lin et al. (2002) compared the EPSCs in two *in vitro* preparations, in dissociated cultures extracted from rat embryos (E18) and in dissociated cultures from P0 donors. At 7 DIV, the EPSCs in E18 cultures were small and almost exclusively mediated by AMPARs, whereas the EPSCs in P0 cultures were larger and mediated by both AMPARs and NMDARs. These results suggest a stronger contribution of NMDARs in P0 cultures and support our observation that NBs remain present in the network activity even after blocking AMPARs. We present here and in the upcoming paragraphs crucial contributions of slow glutamatergic NMDARs to the network activity. These roles have been previously underestimated and thus require futher attention in the future.

In addition to addressing the roles of slow and fast excitatory receptors, we characterized the contribution of fast ionotropic GABA_A_Rs. In networks mediated by both AMPARs and NMDARs, GABA_A_Rs reduced the overall spiking by inhibiting both the initiation and termination phases of NBs. Fast inhibition modulated the spatio-temporal propagation of NBs by reducing the number of active electrodes and at the same time increased the time needed to recruit them into NBs. In order to better understand the mechanisms of interaction between GABA_A_Rs and each type of the considered excitatory receptors, the contribution of GABA_A_Rs was characterized in dominantly AMPAR-mediated and in dominantly NMDAR-mediated networks. In NMDAR-mediated networks, GABA_A_Rs contributed to the dampening of the termination phase of NBs while at the same time reducing the frequency of NB occurrences. In AMPAR-mediated networks, the GABA_A_Rs reduced the overall firing, inhibited the initiation phase of NBs and slowed down the recruitment of electrodes into NBs. The observed changes were more pronounced than those found in NMDAR-mediated networks, suggesting a profound interplay between AMPAR and GABA_A_R receptors, possibly arising from similar fast kinetics. Furthermore, we showed that GABA_A_Rs reduced the overall spiking activity of NBs during both the early and late phases of the NBs, slowed down the network recruitment and spiking during the NBs as well as reduced the number of active electrodes attending to NBs (see Supplementary Figures S1–S3). Some aspects of GABA_A_Rs contribution have been shown previously in cortical *in vitro* networks. It has been shown that the termination phase of NBs substantially increases in intensity and duration after GABA_A_R blocking with known antagonists (including 10 μM BIC, 5/10 μM PTX or 20 μM gabazine) (Jimbo et al., 2000; Weihberger et al., 2013; Baltz and Voigt, 2015; Okujeni et al., 2017). This finding is consistent with our results. However, to the best of our knowledge, the role of GABA_A_Rs in solely AMPAR-mediated or solely NMDAR-mediated networks has not been addressed before *in vitro*.

The striking property of NMDAR- and GABA_A_R-mediated (gradual AMPARs blocked) networks is the emergence of SBs carrying tens of NBs confined within the duration of a SB. The SBs were observed exclusively in the experiments with gradual AMPAR blocking but not in the experiments with acute AMPAR blocking, which suggests different biophysical mechanisms engaged by different blocking protocols. The presence of SBs affected both the global properties of network activity and the fine structure of the spatio-temporal patterns. The overall NB frequency, which significantly decreased in networks probed by acute AMPAR blocking, was rescued due to the high occurrence of NBs within a SB. The similarity in spatio-temporal patterns within NBs increased after gradual blocking of AMPARs and the emergence of SBs. This increased similarity in spatio-temporal patterns might be due to the fine tuning of inhibition-excitation balance in networks with gradual AMPAR blocking. Compared to acute blocking, the gradual blocking protocols provided more flexibility in manipulating the activation of synaptic receptors and allowed progressive changing of the excitation-inhibition balance. Thus, it is possible that the gradual protocol left a sufficient amount of active AMPARs that further activated inhibitory receptors and increased the overall inhibition to excitation ratio. This is supported by studies where the inhibition-excitation balance was manipulated through an increase in the total number of interneurons in cultures and identified the same type of SBs in network activity (Chen and Dzakpasu, 2010). Another mechanism capable of reducing the variability in spatio-temporal patterns is a reduction in the number of burst initiation zones. Previous studies have demonstrated that cortical and hippocampal networks *in vitro* tend to develop a number of localized areas that promote NB initiation (Soriano et al., 2008; Okujeni et al., 2017). Gradual AMPAR blocking reduces the number of these burst initiation zones (according to the preliminary data analysis done to quantify NB similarity) which effectively increases the similarity between observed NBs. An analogous increase in NB similarity was seen in the study that effectively reduced the number of burst initiation zones by inhibiting the protein kinase C (PKC) in postnatal cortical cultures (Okujeni et al., 2017). Additionally, PKC is known to enhance AMPAR conductance via phosphorylation of the AMPAR subunit (Jenkins and Traynelis, 2012). Therefore, the modulations of overall network activity seen after the inhibition of PKC most likely correlate with the effects obtained by AMPAR blocking. Various other cellular mechanisms and phenomena may also have an influence on the observed SB dynamics *in vitro*, including synaptic scaling (Fong et al., 2015) and AMPAR/NMDAR trafficking (Sheng and Lee, 2001; Carroll and Zukin, 2002), extrasynaptic receptors (Parri et al., 2001), astrocytes (Bazargani and Attwell, 2016), dendritic properties and NMDA spikes (Palmer et al., 2014) and even ephaptic coupling (Martinez-Banaclocha, 2018). Further addressing of these mechanisms is beyond the scope of our study.

We presented an extensive collection of experimental protocols designed to carefully manipulate the balance of excitatory and inhibitory receptors by blocking specific synaptic mechanisms and thereby analyze the consequences on network-level activity. Further research on the mechanisms behind NBs will benefit from combining experimental with computational techniques. For instance, with computational models we can further study the cellular and synaptic mechanisms as well as the structural organization of neuronal networks that best reproduce the experimental data. In addition, a well-defined data-driven computational model could reduce the amount of biological experiments and thus accelerate scientific progress. Several computational studies have addressed selected mechanisms affecting the characteristic features of MEA-recorded NBs (Giugliano et al., 2004; Baltz et al., 2011; Gigante et al., 2015; Lonardoni et al., 2017; Manninen et al., 2018). Typically, these studies rely on phenomenological network models and show qualitative agreement with network-level experimental data. Recent advances in neuroinformatics tools (Davison et al., 2009; Denker and Grün, 2016) and infrastructure (Amunts et al., 2016) provide an opportunity to expand these studies by constructing more complex models incorporating details of underlying biophysical mechanisms. Such models can be carefully constrained using multiple modalities of experimental data (e.g., patch-clamp recordings in addition to the MEA recordings considered here) and computationally intensive data-driven modeling protocols. However, the majority of experimental studies are not designed to support computational modeling. Experimental results are often not presented in a manner that supports easy extraction of quantitative values needed for computational modeling. The presented results are compressed and often lack crucial information. In this paper we would like to suggest a different, model friendly manner for presenting experimental data: we carefully quantified different phases and aspects of network activity, provided statistics for them, and listed the obtained quantitative values. Information about the extensive data analysis that we performed is used to support ongoing modeling efforts (Aćimović et al., manuscript in preparation).

This study concludes that the fast AMPARs have a dominant role in the initiation of NBs by rapidly recruiting neurons and that the slow NMDARs maintain the elevated NBs. GABA_A_Rs strongly inhibit the AMPAR-mediated spiking and further dampen the NMDAR-mediated termination phase. According to our study, there is an active interplay between the fast GABA_A_R- and fast AMPAR-mediated activities. In the gradual blocking of AMPARs, the contribution of the network activity is dominated by the SBs that are composed of tens of slow NMDAR-mediated NBs with highly repetitive spatio-temporal patterns. This phenomenon that has been for the first time shown and carefully quantified in this study requires further attention. Combining experimental wet-lab and *in silico* modeling are required to unravel the roles of the considered mechanisms and their contribution to the network level activity.

## Data Availability

The datasets for this study will be available in https://github.com/HTeppola/Front_Cell_Neurosci_Network_Burst_Analysis. Requests to access the datasets should be directed to the corresponding author, M-LL. Any use and subsequent publication of the data presented in this publication and repository must be cited accordingly (i.e., include citation to this article and to the GitHub repository).

## Author Contributions

HT and M-LL conceived the study presented in this manuscript. HT organized the data that she previously recorded, analyzed, and presented in Teppola et al. (2011), reprocessed and re-analyzed the data to support better fitting of computational modeling of cell culture dynamics, performed data-analysis, prepared the figures, and drafted the manuscript. HT, JA, and M-LL selected the data analysis procedures and new code implementing those procedures, jointly interpreted and discussed the results and their implications, revised the manuscript, and approved the final version of the manuscript. JA and M-LL reviewed the measures, statistical analysis, and result presentation to select those that best support data-driven computational modeling.

## Conflict of Interest Statement

The authors declare that the research was conducted in the absence of any commercial or financial relationships that could be construed as a potential conflict of interest.

## Abbreviations

AMPA: alpha-amino-3-hydroxy-5-methyl-4-isoxazolepropionic acid
APV: 2-amino-5-phosphovaleric acid
BF: burst frequency
BIC: bicuculline
BL: burst length
BS: burst size
CC: correlation coefficient
CNQX: 6-cyano-7-nitroquinoxaline-2,3-dione
CO_2_: carbon dioxide
CPP: (±)-3-(2-Carboxypiperazin-4-yl)propyl-1-phosphonic acid
CTRL, control: D-AP 5, D-2-Amino-5-phosphonovaleric acid
DIV: days *in vitro*
E18: embryonic day 18
EPSC: excitatory postsynaptic current
FP: falling phase
FR: firing rate
GABA: gamma-aminobutyric acid
IBI: interburst interval
ISI: inter-spike interval
MEA: microelectrode array
MEM: minimum essential medium
MFR: maximal firing rate
Mg^2+^: magnesium
NB: network burst
NBQX: 1,2,3,4-Tetrahydro-6-nitro-2,3-dioxo-benzo[f]quinoxaline-7-sulfonamide disodium salt hydrate
NMDA: *N*-Methyl -D-aspartic acid
OFR: overall firing rate
P0: postnatal day 0
PBS: phosphate-buffered saline
PKC: protein kinase C
PTX: picrotoxin
RC: electrode recruitment count
RP: rising phase
SB: super burst
SD: standard deviation.
