# Supplementary material for: Unique Features of Network Bursts Emerge From the Complex Interplay of Excitatory and Inhibitory Receptors in Rat Neocortical Networks
Source: Front Cell Neurosci. 2019 Sep 6;13:377. doi: 10.3389/fncel.2019.00377 (PMC6742722; doi:10.3389/fncel.2019.00377)
Supplement: Supplementary file 1 [file Data_Sheet_1.pdf]

Materials and Methods in *Supplementary material* section:

Pharmacology:

In pure gradual disinhibition the selected amount of PTX (0.1µM, 0.2µM, 0.3µM, 1µM, 2µM, 3µM) was applied to antagonize the $GABA_AR$-mediated ionotropic GABAergic transmission (Figure S1A, S1B, Table S1, Protocol id s1). In the case of gradual disinhibition after the acute application of excitatory receptor antagonist (AMPAR or NMDAR complete blockade), an increasing amount of PTX was gradually applied to first partially (0.1µM, 1µM) and then completely block the $GABA_AR$-mediated transmission (10µM, 40µM) (Figure S1C, S1D, Table S1, Protocol id s2, s3).

Experimental protocol:

In the case of gradual disinhibition ($GABA_AR$ blockade), a total of three cell cultures from two different preparations were studied. One culture was used solely for performing experiments on the gradual disinhibition of $GABA_AR$ (0.1, 0.2, 0.3, 1, 2, 3µM PTX). Two cultures were used for performing experiments on the gradual disinhibition of $GABA_AR$ (0.1, 1, 10, 40µM PTX) either after the acute AMPAR blockade (30µM NBQX) or the acute NMDAR blockade (30µM D-AP5). The experiments consisted of the following steps: **(1)** 25 min-long CTRL recording, **(2)** acute application of 30µM excitatory antagonist (NBQX in experiments with AMPAR blockade, D-AP5 in experiments with NMDAR blockade), **(3)** recording for 25min, **(4)** application of 0.1µM $GABA_AR$ antagonist (PTX), **(5)** recording for 25min, **(6)** application of 1µM PTX, **(7)** recording for 25min, **(8)** application of 10µM PTX, **(9)** recording for 25min, **(10)** application of 40µM PTX, and **(11)** recording for 25min (Figure S1C, Table s1, Protocol id s2, s3). After the application of the antagonist, the first ten minutes of the recording were not analyzed to avoid transition phases. The remaining 15 minutes of the recording were analyzed.

Relative overall firing rate and burst frequency computations:

The relative overall firing rate (OFR, [Hz]) and network burst frequency (BF, (NB/min)) were computed for each recording and then displayed as separate graph representations of the rates in in the cases of gradual application of the inhibitory receptor blockade (see Table s1, Protocol id s1, s2, s3) (Figures S1B, S1D). To compute the relative OFR, the number of all spikes was divided by the duration of the recording period in seconds. To compute the BF, the number of NBs was divided by the duration of the recording period in minutes. The values were normalized to CTRL condition by dividing the value with the CTRL value.

Characterization of burst measures:

For gradual disinhibition, burst measures were not normalized to CTRL and were displayed as a box plot representation of the pooled measures. Box plots show the median, 25th and 75th percentiles with whiskers extending to the minimal and maximal values, and the plus signs represent outliers (Figure S2B). Wilcoxon rank sum test and p-values were computed for all pooled burst measures between CTRL and each condition. Differences were considered to be significant when $p_{ranksum} < 0.001$. This significance is indicated with * in Figure S2B.

Interspike interval and interburst interval distributions:

The mean of ISI distributions from all cultures of the same condition were computed and the values of i were plotted on the x-axis with a logarithmic scale (log(TIME[sec]). Statistical analysis was performed for all ISIs between each condition in the same culture using Wilcoxon rank sum test and p-values. If the tests showed a similar result for the culture the differences were considered significant and were displayed as $***p_{ranksum} < 0.001$, $**p_{ranksum} < 0.01$, $*p_{ranksum} < 0.05$ in Figure S3A.

The IBI distributions were computed and the values of i were plotted on the x-axis with a logarithmic scale. Statistical analysis was performed for all IBIs between each condition in the same culture using Wilcoxon rank sum test and p-values. If the tests showed a similar result for the culture, the differences were considered significant and were displayed as $***p_{ranksum} < 0.001$, $**p_{ranksum} < 0.01$, $*p_{ranksum} < 0.05$ in Figures S3B.

Network recruitment time computed as cumulative relative number of active electrodes:

Cumulative number of electrodes recruited at the onset of a NB, relative to the CTRL, was computed for each condition and each culture. For each NB, the timing of the first spike at each electrode was stored. These stored spike times were used to compute a time vector as follows. We discretized the NB duration, starting at 0.5 s after the onset of NB and using the 0.0001 s discretization time step. For each discrete time step we counted the number of electrodes activated until that time, the obtained cumulative number of electrodes was stored into the time vector. Next, we pooled all the time vectors representing all NBs recorded from the same culture and condition. The mean value of activated electrodes was computed for each time step, by averaging over all NBs. The obtained results illustrate the speed of electrode recruitment, and are shown in Supplementary Figure S3C.

Similarity analysis of the activity propagation patterns:

Statistical analysis was performed for all CCs between each condition in the same culture using Wilcoxon rank sum test and p-values. If the tests showed a similar result for the culture the differences were considered significant and was displayed as $***p_{ranksum} < 0.001$, $**p_{ranksum} < 0.01$, $*p_{ranksum} < 0.05$ in Figures S4B.

Results in *Supplementary material* section:

Quantitative characterization of $GABA_A$Rs: reduced overall spiking and slowing down the activity propagation

The fast ionotropic $GABA_A$Rs are the main mediator of the inhibitory activity in neuronal networks beside the metabotropic $GABA_B$Rs. The $GABA_A$Rs contributed to the overall network excitability via reduction of network activity. In the temporal domain, the $GABA_A$Rs reduced the overall spiking activity of NBs during both the early and late phases of the NBs as well as slowed down the activity propagation and spiking during the NBs. In the spatial domain, $GABA_A$Rs effectively reduced the number of active electrodes attending to NBs.

In order to study the influence of GABA$_A$Rs on network activity dynamics, the networks were gradually disinhibited in a concentration-dependent manner by applying increasing concentrations of picrotoxin (0.1, 0.2, 0.3, 1, 2, and 3µM PTX, the total added PTX concentration was 6.6µM). Our results showed that the OFR gradually increased to almost four-fold while BF increased only mildly in comparison to the CTRL condition (Figure S1B). When applying 2µM (the total concentration was 3.6µM) and 3µM (in total 6.6µM) of PTX, the NB measures including the BL, FP, MFR, BS and RC significantly increased ($p < 0.001$) when compared to the CTRL condition (Figure S2B (**top row**)). The rising phase significantly decreased ($p < 0.001$) when applying 0.3, 1, 2 and 3µM PTX in comparison to the CTRL condition (Figure S2B (**top row**)). The duration of ISIs significantly decreased ($p < 0.001$) with the PTX in comparison to the CTRL condition (Figure S3A (**left panel**)), indicating more frequent spiking activity in disinhibited networks. The recruitment time of neurons at the beginning of the NBs gradually decreased by disinhibition, meaning faster recruitment of activity (Figure S3C (**leftmost panel**)). The results of a similarity analysis of the activity propagation patterns indicate that the gradual GABA$_A$R blockade significantly increased ($p_{\text{ranksum}} < 0.001$) the similarity (Figure S4A, S4B).

GABA$_A$Rs dampen the termination phase of network bursts and decrease the burst frequency in the NMDAR-mediated networks:

To assess how GABA$_A$ receptors shape the NMDAR-mediated spontaneous NB dynamics, first the AMPARs were acutely blocked (30µM NBQX, i.e. NMDAR-mediated networks), and then the networks were gradually disinhibited by applying 0.1, 1, 10 and 40µM PTX (the total added PTX concentration was 51.1µM). After AMPAR blocking (30µM of NBQX), the OFR dropped to about half of the control value and BF almost to zero in comparison to the CTRL condition. Further gradual disinhibition (with 0.1, 1, 10 and 40µM of PTX) brings the OFR to levels comparable to or higher than the CTRL condition, reaching about 1.7 of the CTRL value when disinhibited with the highest concentration of PTX. In addition, disinhibition restored the BF to about half of the control value (Figure S1D). However, disinhibition did not increase the OFR or BF in the NMDAR-mediated networks as much as in the AMPAR-mediated networks (30µM D-AP5) or in the solely disinhibited networks (Figures S1B, S1D). The results indicate that AMPARs are crucial for the higher firing rate within the NBs when networks were disinhibited and that NMDAR-mediated spiking was not similarly inhibited as AMPAR-mediated spiking by GABA$_A$Rs (Figure S1B, S1D). A more detailed analysis of the burst measures showed that the NMDAR-mediated BL, FP and BS decreased when networks were disinhibited in comparison to inhibited networks, meaning that GABA$_A$Rs dampened the termination of NBs (Figures S2A, S2B (**middle row**)). The number of active electrodes significantly increased ($p < 0.001$) by disinhibition in NMDAR-mediated networks in comparison to the CTRL condition. This result indicates that the disinhibition increased the spatial coverage of network activity in NMDAR-mediated networks (Figure S2B (**rightmost panel in middle row**)). The AMPAR blockade (30µM NBQX) significantly prolonged the ISIs (Figure S3A (**middle panel**)). Although the disinhibition shortened the ISIs in comparison to the AMPAR blocked condition, the ISIs were still significantly longer ($p < 0.001$) after disinhibition when compared to the CTRL condition (Figure S3A (**middle panel**)). These results demonstrate that disinhibition is not able to significantly increase spiking frequency within the NMDAR-mediated NBs (Figure S3A (**middle panel**)). The gradual disinhibition significantly shortened ($p < 0.001$) the IBIs when compared to the AMPAR blocked condition (Figure S3B (**middle panel**)). The recruitment time of neurons increased in NMDAR-

mediated networks and began to decrease when the networks were further disinhibited (Figure S3C (**middle panel**)). However, disinhibition in NMDAR-mediated NBs did not shorten the recruitment time to the level of the CTRL condition, assuring that AMPARs are crucial for the fast activity propagation at the beginning of the NBs.

GABA$_A$Rs contribute to inhibiting the spiking frequency and preventing the fast spread of activity propagation in the AMPAR-mediated networks:

In order to study the influence of GABA$_A$ receptors on shaping the AMPAR-mediated NB activity dynamics, the NMDARs (30µM D-AP5) were acutely blocked and continued to gradually disinhibit the networks by applying 0.1, 1, 10 and 40µM of PTX (the total added PTX concentration was 51.1µM). The results showed that the total gradual disinhibition in the AMPAR-mediated networks (30µM D-AP5) increased the OFR over threefold and BF only mildly, similarly to solely disinhibited cultures (Figure S1D (**black lines**)). A more detailed analysis of the burst measures demonstrated that the BL, FP, RP, MFR, BS and RC significantly decreased ($p < 0.001$) in AMPAR-mediated networks in comparison to the CTRL condition (Figure S2B (**bottom row**)). The gradual disinhibition did not significantly increase the BL, FP or RP in the AMPAR-mediated networks in comparison to the CTRL condition (Figure S2B (**bottom row**)). The antagonist of the NMDARs effectively removed the late phase of network burst profiles. The late phase was not restored by subsequent disinhibition (Figure S2A (**bottom row**), S2B (**bottom row**)). Furthermore, disinhibition significantly increased ($p < 0.001$) the MFR, BS and RC in AMPAR-mediated networks in comparison to the CTRL condition, meaning that GABA$_A$Rs strongly inhibited the AMPAR-mediated spiking within the NBs (Figure S2B (**bottom row**)). The ISI distributions did not change by NMDAR blockade (30µM D-AP5) (Figure S3A (**left panel**)). Disinhibition significantly shortened ($p < 0.001$) the ISIs only when using as high concentrations as 10µM and 40µM of PTX (Figure S3A (**left panel**)), suggesting important interplay between the GABA$_A$Rs-mediated inhibition and the AMPAR-mediated fast spiking. The NMDAR blockade (30µM D-AP5) increased the IBIs, but disinhibition did not change them in AMPAR-mediated networks in comparison to the CTRL condition (Figure S3B (**left panel**)). The recruitment time of neurons decreased when blocking the NMDARs and continued to decrease when gradually applying increasing concentrations of PTX (Figure S3C (**rightmost panel**)), meaning that NMDARs and GABA$_A$Rs slow down the network recruitment.

Table S1. List of supplemental protocols including recording conditions, blocked and functional receptors and the number of used cultures.

**Protocol id**	**Recording condition/ Drug**	**Concentration of an antagonist**	**Blocked receptors**	**Functional receptors among the considered ones**	**Number of cultures**
**Gradual disinhibition**					
s1	CTRL<br>PTX	-<br>.1, .2, .3, 1, 2, 3μM (in total 6.6μM)	None<br>$GABA_A$	All<br>AMPA, NMDA	1
**Gradual disinhibition after the acute application of the excitatory receptor antagonists**					
s2	CTRL<br>NBQX<br>PTX	-<br>30μM<br>.1, 1, 10, 40μM (in total 51.1μM)	None<br>AMPA<br>$GABA_A$	All<br>NMDA, $GABA_A$<br>NMDA	1
S3	CTRL<br>D-AP5<br>PTX	-<br>30μM<br>.1, 1, 10, 40μM (in total 51.1μM)	None<br>NMDA<br>$GABA_A$	All<br>AMPA, $GABA_A$<br>AMPA	1

Supplementary Figures:

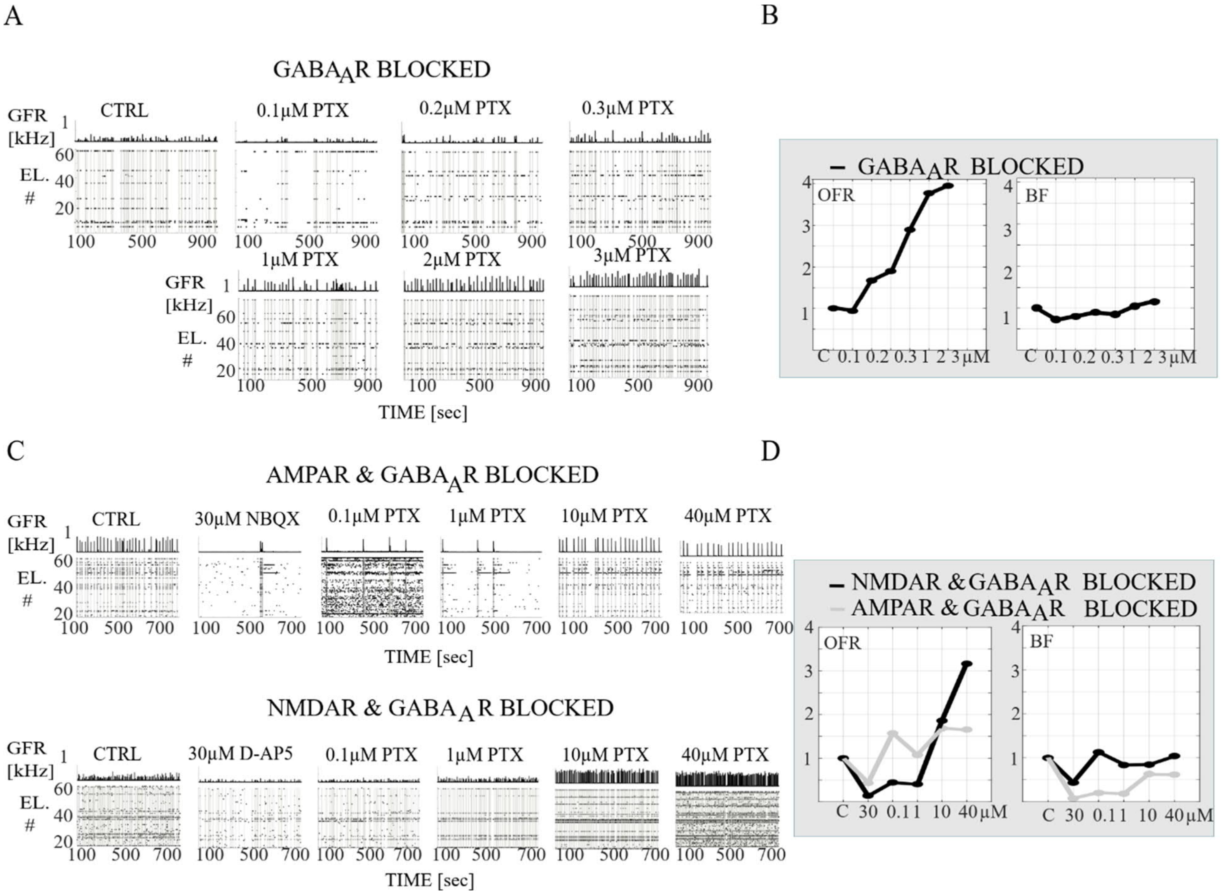

S1. Receptor-dependent network-wide activity probed by **gradual disinhibition**, relative changes in OFR [Hz] and in BF (NB/min). **(A)** and **(C)** Raster plots of network activity of the CTRL recording probed by three different types of gradual application of $GABA_ARs$ antagonist (PTX). In each subpanel, the global firing rates (GFR, [kHz]) are displayed on top of raster plots of spike times [sec] from electrodes (EL #) in each condition. **(A)** The recording period of 900 seconds from network probed by a gradual application of $GABA_AR$ antagonist (0.1, 0.2, 0.3, 1, 2, and 3μM PTX). **(C)** The top row shows the recording from the network that is probed first by an acute application of AMPAR antagonist (30μM NBQX) and then by a gradual application of $GABA_AR$ antagonist (0.1, 1, 10, and 40μM PTX). The bottom row shows the recording from the network that is probed first by an acute application of NMDAR antagonist (30μM D-AP5), and then by a gradual application of $GABA_AR$ antagonist (0.1, 1, 10, and 40μM PTX). **(B)** and **(D)** Relative changes in OFR and in BF with respect to those obtained from the CTRL condition as shown for each recording. The relative change is computed as described in the *Materials and Methods* section. The values of the applied concentrations of each antagonist are shown on the x-axis. The disinhibition of the CTRL culture increased the OFR, but only slightly the BF **(B)**. Acute 30μM AMPAR and NMDAR antagonists decreased the OFR and BF **(D)**. An acute application of AMPAR antagonist decreased the BF more than the NMDAR antagonist **(D)**. In contrast, an acute application of NMDAR antagonist decreased the OFR more than AMPAR antagonist **(D)**. Pure disinhibition increased the OFR four fold **(B)**. The disinhibition of the NMDAR blocked culture

increased the OFR more than three fold **(black line in D)** similarly to pure disinhibition **(B)**. However, the disinhibition of the AMPAR blocked culture increased the OFR only 1.6 fold **(grey line in D)**.

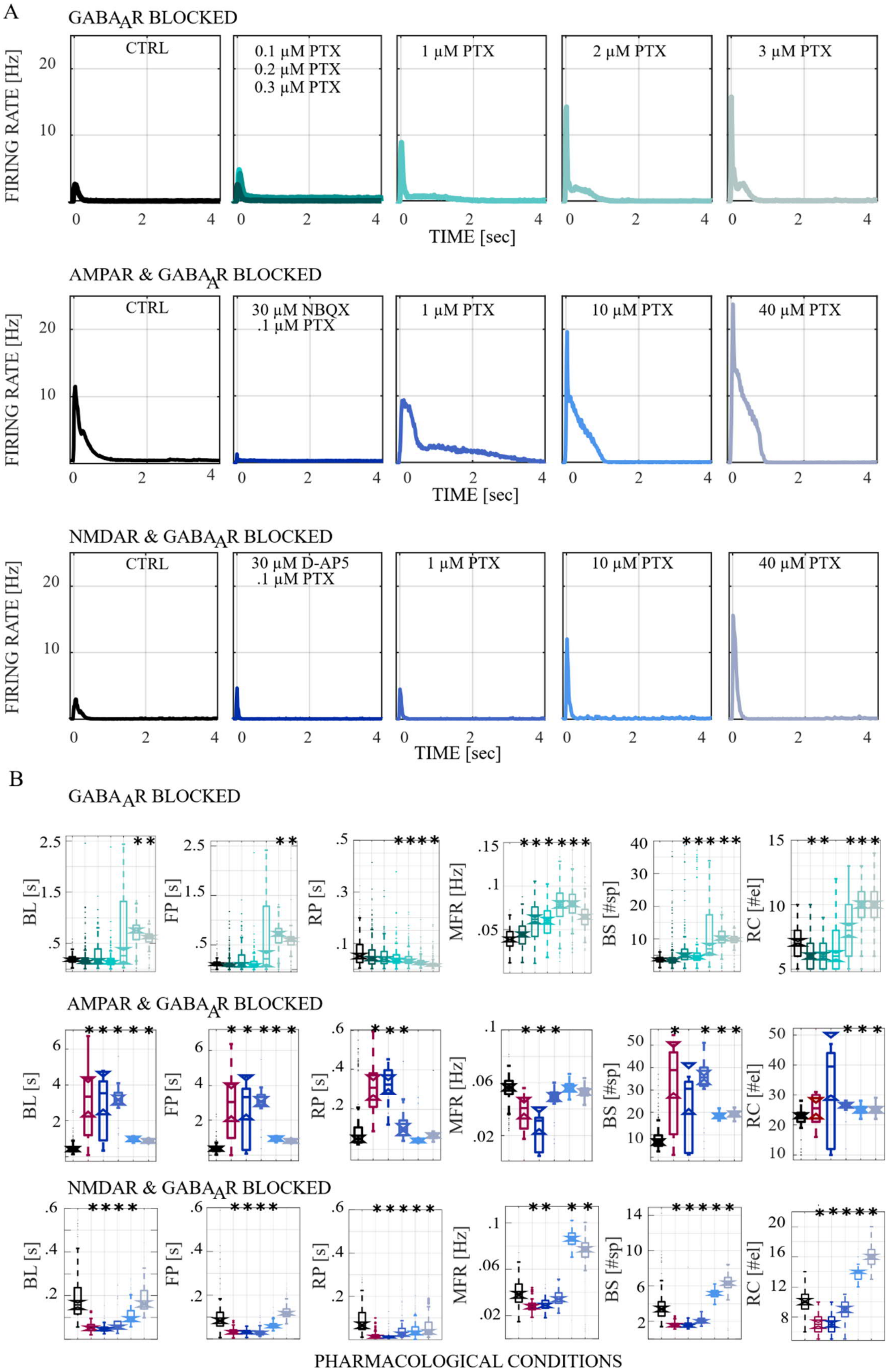
A
GABAAR BLOCKED
CTRL
0.1 µM PTX
0.2 µM PTX
0.3 µM PTX
1 µM PTX
2 µM PTX
3 µM PTX
FIRING RATE [Hz]
TIME [sec]
AMPAR & GABAAR BLOCKED
CTRL
30 µM NBQX
.1 µM PTX
1 µM PTX
10 µM PTX
40 µM PTX
NMDAR & GABAAR BLOCKED
CTRL
30 µM D-AP5
.1 µM PTX
1 µM PTX
10 µM PTX
40 µM PTX
B
GABAAR BLOCKED
BL [s]
FP [s]
RP [s]
MFR [Hz]
BS [#sp]
RC [#el]
AMPAR & GABAAR BLOCKED
NMDAR & GABAAR BLOCKED
PHARMACOLOGICAL CONDITIONS

S2. The influence of **gradual disinhibition** on network burst profiles and burst measures. **(A)** The top row compares the burst profiles [Hz] of CTRL data to the data collected after blocking $GABA_AR$s with .1, .2, .3, 1, 2 and 3µM PTX. The middle row compares the CTRL with the data collected after acutely antagonized AMPARs (30µM NBQX) and gradually antagonizing $GABA_AR$s (.1, 1, 10 and 40µM PTX). The bottom row shows the similar comparison as the middle row, except that in this case NMDARs are acutely antagonized (30µM D-AP5) and $GABA_AR$s are again gradually antagonized (.1, 1, 10, and 40µM PTX). The presented profiles are computed using the spike-data from Figure S.1. The colors on the A and B panels corresponds to each condition and indicates the applied concentration of an antagonist. **(B)** The changes of characteristic burst measures computed from burst profiles are shown in **(A)**, including BL, FP, RP, MFR, BS and RC (see *Materials and Methods*). The top row shows the data from the experiment where the $GABA_AR$s are gradually blocked, the middle row shows acute AMPAR blockade with gradual $GABA_AR$ blockade and the bottom row acute NMDAR blockade with gradual $GABA_AR$ blockade, similar to **(A)**. Each box plot represents the median, 25th and 75th percentiles and whiskers extending to minimal and maximal values. **Top row**; BL, FP and BS significantly increased ($p_{\mathrm{ranksum}} < 0.001$) when using 2 and 3µM PTX compared to CTRL. RP significantly decreased ($p_{\mathrm{ranksum}} < 0.001$) when applying .3, 1, 2 and 3µM PTX, MFR significantly increased ($p_{\mathrm{ranksum}} < 0.001$) with all concentrations of PTX and RC first significantly decreased ($p_{\mathrm{ranksum}} < 0.001$) when using .1 and .2µM PTX and then significantly increased ($p_{\mathrm{ranksum}} < 0.001$) when applying 1, 2 and 3µM PTX. **Middle row**; BL, and FP significantly increased ($p_{\mathrm{ranksum}} < 0.001$) first when using 30µM NBQX and then when applying .1, 1, 10 or 40µM PTX compared to CTRL. However, BL, FP, RP and BS remarkably decreased with 10 and 40µM PTX when compared to lower PTX concentrations. RP significantly increased and MFR significantly decreased ($p_{\mathrm{ranksum}} < 0.001$) when using 30µM NBQX and when applying .1 and 1µM PTX. BS significantly increased ($p_{\mathrm{ranksum}} < 0.001$) when using 30µM NBQX and when applying 1, 10 and 40µM PTX. RC significantly increased when using 1, 2 and 3µM PTX. **Bottom row**: BL and FP significantly decreased after 30µM D-AP5 and after .1, 1 and 10µM disinhibition. However, in contrast to the AMPAR and $GABA_AR$ blocked case BL and FP increased by growing disinhibition with NMDAR antagonist. RP also significantly decreased ($p_{\mathrm{ranksum}} < 0.001$) in all conditions compared to CTRL. MFR, BS and RC significantly decreased ($p_{\mathrm{ranksum}} < 0.001$) when applying 30µM D-AP5 and when using .1µM PTX. On the contrary, MFR, BS and RC significantly increased ($p_{\mathrm{ranksum}} < 0.001$) when PTX was increased to 10 and 40µM. Interestingly, burst measures change in opposite directions depending on whether AMPAR or NMDAR blockade is involved. In addition, disinhibition alters the burst measures in opposite directions depending on whether AMPARs or NMDARs are blocked first. *$p_{ranksum} < 0.001$.

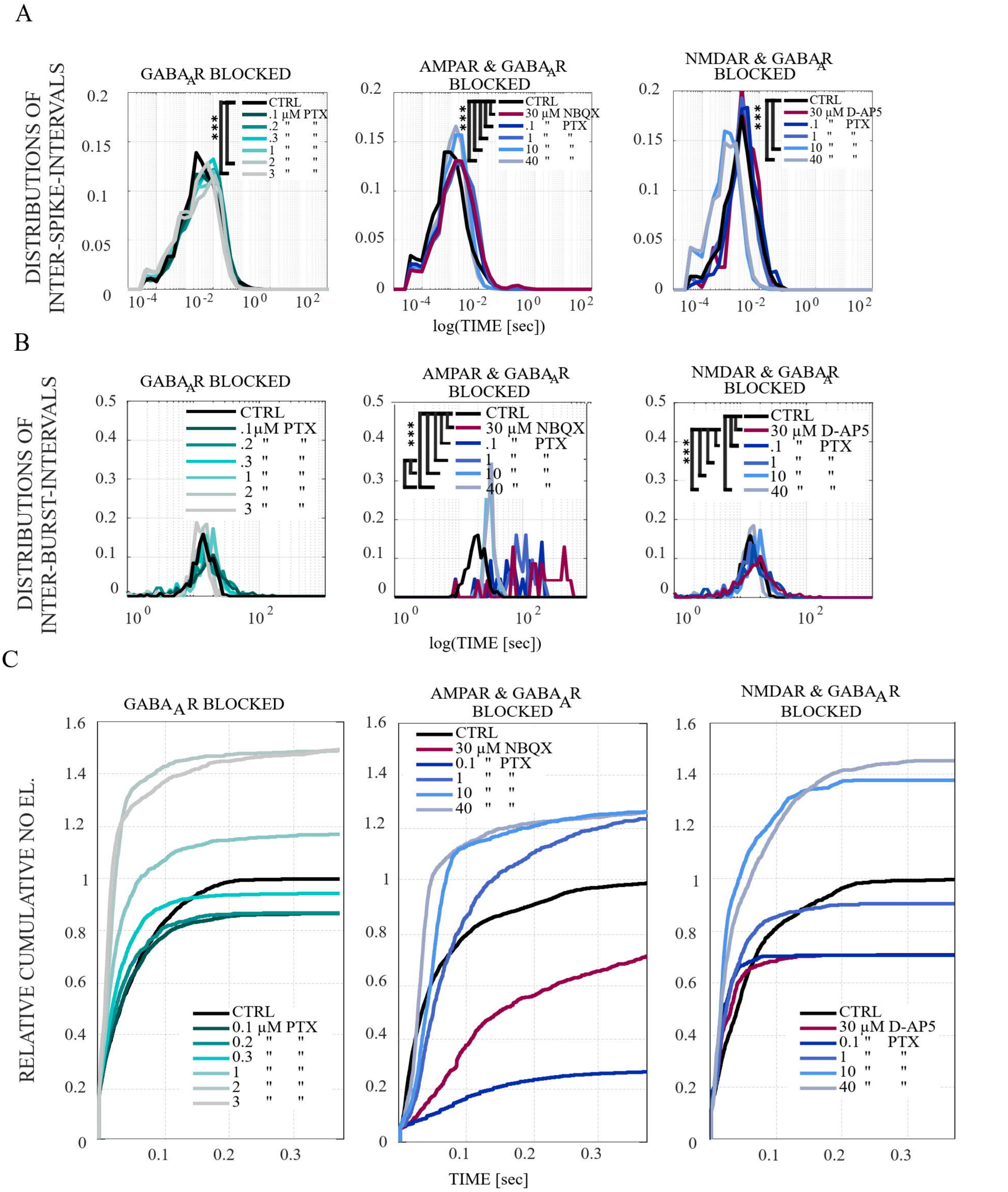

S3. (A) The excitatory and inhibitory receptor dependence on interspike intervals within NBs. Neurons in gradually disinhibited network first expressed significantly shorter ISIs when applying 2 and 3µM PTX ($p_{ranksum} < 0.001$) compared to CTRL **(left panel).** The acute application of 30µM NBQX significantly increased ISIs ($p_{ranksum} < 0.001$) similarly to the gradual blockade of AMPARs in comparison to the CTRL condition. Gradual disinhibition with .1, 1, 10 and 40 µM PTX

gradually decreased ISIs in comparison to the condition with AMPAR blockade (30µM NBQX). However, the fractions of ISI distributions were still significantly higher in comparison to the CTRL condition ($p_{\text{ranksum}} < 0.001$) **(middlet panel).** ISI distribution did not differ in an acutely NMDAR blocked network (30µM D-AP5). Interestingly, disinhibition with 10 and 40µM PTX clearly shifts ISI distributions towards the lower fractions in a NMDAR and $GABA_AR$ blocked network, meaning significantly shorter distances between spikes within NBs in neurons without functional NMDARs and $GABA_ARs$ ($p_{\text{ranksum}} < 0.001$) **(right panel)**. **(B)** A change in the excitatory and inhibitory receptor balance modulates the duration of interburst intervals and thus the frequency of NB events. Neurons in gradually disinhibited networks expressed significantly shorter IBIs than in CTRL networks when the concentration of PTX was 3µM ($p_{\text{ranksum}} < 0.05$) **(left panel)**. A significantly longer fraction of intervals was computed after acute AMPAR blockade (30µM NBQX) and with all concentrations of PTX (.1, 1, 10 and 40µM) compared to CTRL ($p_{\text{ranksum}} < 0.001$) **(middle panel)**. Disinhibition with 10 and 40µM PTX after acute NMDAR blockade (30µM D-AP5) shifted the IBI distributions to lower fractions compared to CTRL ($p_{\text{ranksum}} < 0.001$), meaning shorter distances between NBs **(right panel)**. Wilcoxon rank sum test and p-values were computed for all ISIs and IBIs in each condition and in each culture. If the tests showed similar results and p-values for every culture, the results were displayed in **(A)**, and **(B)**. $^{***}p_{\text{ranksum}} < 0.001$, $^{**}p_{\text{ranksum}} < 0.01$, $^{*}p_{\text{ranksum}} < 0.05$. The x-scale is logarithmic. **(C)** A change in the excitatory and inhibitory receptor balance modulates network-wide electrode recruitment speed at the beginning of the bursts. Gradual disinhibition by $GABA_AR$ blockade remarkably sped up the network recruitment speed as well as increased the number of active electrodes in all three cultures with increasing concentrations of the $GABA_AR$ antagonist PTX. The first example on the left shows the increasing influence of the gradual application of the $GABA_ARs$ antagonist (0.1, 0.2, 0.3, 1, 2, 3µM PTX) on the electrode recruitment speed **(left panel)**. The second, middle example shows that the acute AMPAR blockade (30µM NBQX) first slows down the electrode recruitment speed with less active electrodes, and the gradual disinhibition with PTX (0.1, 1, 10, 40µM) accelerated it, starting with 1µM PTX in comparison to the AMPAR blocked condition (middle panel). An acute NMDAR blockade with D-AP5 (30µM) sped up the electrode recruitment speed with less active electrodes in comparison to the CTRL condition. The gradual disinhibition with PTX (0.1, 1, 10, 40µM) gradually increased the number of active electrodes and further accelerated the recruitment speed **(right panel).** The disinhibition of the network by the $GABA_AR$ antagonist by gradual **(C)** application increased the recruitment speed even more than the NMDAR blockade. Values were normalized to the CTRL conditions. Bin width is 0.1ms.

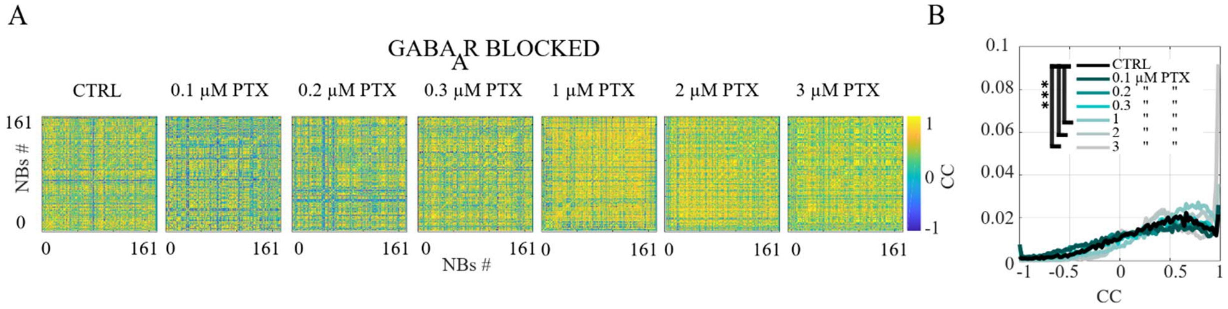

S4. (A) Correlation coefficient (CC) matrices computed for pairwise-spike-time-difference matrices between each NB in each condition of gradual applications. **(B)** Mean distributions of CC across the NBs and networks are shown. The gradual $GABA_AR$ blockade changed slightly the CC matrices (N = 161 NBs). The gradual $GABA_AR$ blockade with 1, 2, and 3µM PTX significantly increased ($p_{ranksum} < 0.001$) the similarity between spatio-temporal patterns in comparison to the CTRL condition**.**
